# Ligands Mediate Anion Exchange between Colloidal Lead-Halide Perovskite Nanocrystals

**DOI:** 10.1021/acs.nanolett.2c00611

**Published:** 2022-05-23

**Authors:** Einav Scharf, Franziska Krieg, Orian Elimelech, Meirav Oded, Adar Levi, Dmitry N. Dirin, Maksym V. Kovalenko, Uri Banin

**Affiliations:** † The Institute of Chemistry and the Center for Nanoscience and Nanotechnology, The Hebrew University of Jerusalem, Jerusalem 91904, Israel; ‡ Institute of Inorganic Chemistry, Department of Chemistry and Applied Bioscience, ETH Zürich, Vladimir Prelog Weg 1, Zürich CH-8093, Switzerland; § Laboratory for Thin Films and Photovoltaics, Empa − Swiss Federal Laboratories for Materials Science and Technology, Ueberlandstrasse 129, Dübendorf CH-8600, Switzerland

**Keywords:** perovskite nanocrystals, anion exchange, kinetics, surface ligands

## Abstract

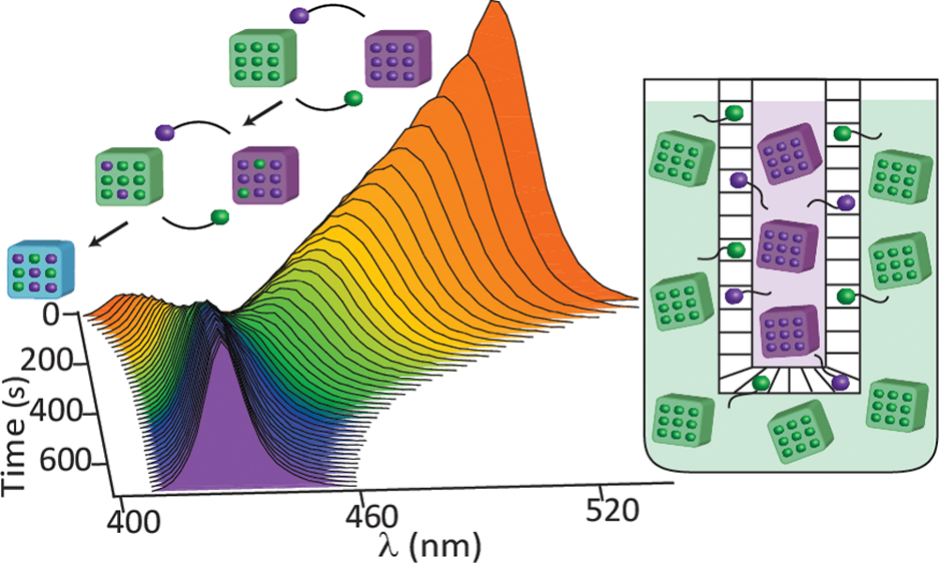

The soft lattice of lead-halide perovskite nanocrystals (NCs) allows tuning their optoelectronic characteristics via anion exchange by introducing halide salts to a solution of perovskite NCs. Similarly, cross-anion exchange can occur upon mixing NCs of different perovskite halides. This process, though, is detrimental for applications requiring perovskite NCs with different halides in close proximity. We study the effects of various stabilizing surface ligands on the kinetics of the cross-anion exchange reaction, comparing zwitterionic and ionic ligands. The kinetic analysis, inspired by the “cage effect” for solution reactions, showcases a mechanism where the surface capping ligands act as anion carriers that diffuse to the NC surface, forming an encounter pair enclosed by the surrounding ligands that initiates the anion exchange process. The zwitterionic ligands considerably slow down the cross-anion exchange process, and while they do not fully inhibit it, they confer improved stability alongside enhanced solubility relevant for various applications.

Inorganic cesium lead-halide perovskite nanocrystals (CsPbX_3_ NCs, X = Cl, Br, I) manifest exceptional optoelectronic properties, in particular, strong emission with a narrow bandwidth.^1−3^ This, alongside their facile synthesis and high defect tolerance, has already led to the demonstration of highly efficient light-emitting diodes,^4−6^ displays,^5^ photodetectors,^7,8^ and solar cells.^4,9−11^ Their low lattice energy and the high concentration of vacancies result in a labile lattice structure, manifested by high halide mobility, which enables anion exchange while maintaining the original NC shape.^12^ Hence, bandgap engineering of the NCs can be achieved by altering the halide composition in the lattice.^13^ Halide mobility was also observed between NCs with different halides, demonstrating the feasibility of cross-anion exchange, without an external halide source.^14,15^ Cross-anion exchange occurs spontaneously in solution until compositional equilibrium of the NCs is reached.

Halide migration and diffusion processes were investigated in perovskite nanoplates^17^ and nanowires^18,19^ as well as for anion exchange between colloidal NCs and halide salts in solution.^20−22^ Previous work on micrometer long nanowires established that anion exchange is facilitated by the diffusion of the halides through the lattice via a vacancy-mediated transport, leading to their homogenization.^19^ In NCs, the surface-to-volume ratio increases dramatically, accentuating surface effects. Anion exchange between NCs and halide salts manifests an exponential time dependence of the change in emission energy and hence composition.^20^ A decrease in the rate of the reaction upon addition of excess ligands to the solution was reported.^22^ Only a few studies focused on the kinetics of cross-anion exchange in NCs,^23^ and a complete mechanism has yet to be derived.

Upon utilizing several types of perovskite NCs with different halide compositions, such variations will affect the photoluminescence (PL) spectrum and quantum yield (QY), possibly jeopardizing the device optoelectronic characteristics.^24^ Surface passivation aimed at stabilizing the NCs and suppressing anion exchange has been reported.^25,26^ Recently, zwitterionic molecules were implemented as surface ligands of perovskite NCs, introducing several improvements to the properties and handling of the NCs. They bind tightly to the surface of the NCs and enhance their structural and colloidal stability as a result of the chelate effect.^27^ This increases the yield of the synthesis and the purity of the NCs dispersion, as NCs capped in zwitterionic ligands are more durable to cleaning procedures.^28^ The tight binding of the ligands to the surface enables higher colloidal stability of the NCs, which can accommodate colloidal NCs in a higher concentration than the conventional monoionic ligands.^27,29^

Herein, we study the kinetics of the cross-anion exchange process in perovskite NCs and explore whether zwitterionic ligands will also have a stabilizing effect on the halide lability. We find that the reaction mechanism bears analogy to the classical “cage effect”, in which the anion-ligand complex diffuses to the surface of the NC and exchanges the complexed anion with a surface anion. Zwitterionic ligands are found to slow down the cross-anion exchange kinetics significantly but do not fully hinder the process. The work thus outlines guidelines and challenges for engineering perovskite NC surfaces with improved stability.

To address these open questions, we studied the cross-anion exchange reaction between pure CsPbCl_3_ and CsPbBr_3_ NCs, comparing the effects of different ligands. Two perovskite NCs systems, each capped with a different type of zwitterionic surface ligands, natural soy lecithin, and 3-(*N*,*N*-dimethyl(octadecyl)ammonio)propane-1-sulfonate (C3-ASC18), were compared with NCs capped with the most commonly used monoionic ligands, oleic acid/oleylammonium (OA/OLA). By continuously monitoring the PL spectrum upon the cross-anion exchange progression, we analyzed the reaction kinetics, and derived its mechanism. The influence of the surface coating was also derived. A model, based on the well-known “cage effect” mechanism in solutions, is suggested to explain our findings. The improved understanding of the cross-anion exchange mechanism can lead to better design and fabrication of perovskite-based devices in various fields, ranging from optical to electronic and photovoltaic applications.

For the cross-anion exchange reactions, CsPbCl_3_ and CsPbBr_3_ NCs with three different surface coatings were synthesized (ranging from 6 to 10 nm in size, see Supporting Information for details, Figure S1 for optical and electron microscopy characterization). Figure 1a presents spectra recorded during a typical cross-exchange reaction (scheme in Figure 1b, details in SI sections 5 and 6) between CsPbCl_3_ and CsPbBr_3_ NCs with an 8.5(±0.5):1 halide ratio respectively, coated by C3-ASC18 zwitterionic ligands (SI sections 2–4 for calculations of NC concentrations). The halide ratio was chosen under the consideration that the PL QY of CsPbBr_3_ is higher than that of CsPbCl_3_, and therefore excess of the latter is required for suitable detection and analysis of the PL peaks. At time *t* = 0, two distinct peaks appear in the PL spectrum at 409 and 510 nm, assigned to the chloride- and bromide-composed NCs, respectively. As the reaction progresses, both PL peaks shift toward each other until merging to a single peak at equilibrium (424 nm). Transmission electron microscopy (TEM) images and powder X-ray diffraction (XRD) show that the exchange process maintains the NCs structure (Figures S3 and S4). The PL behavior is thus attributed to the interchanging halide anion content, in agreement with prior studies.^14,15^ At the end point, all NCs possess the same homogeneous halide composition. The PL peak position is consistent with a Cl:Br ratio of 8.5:1. This is consistent with the extracted peak shift in the powder XRD data using Vegard’s law and with the elemental ratio measured on the final product NCs using scanning electron microscopy–energy dispersive X-ray spectroscopy (SEM-EDS, SI section 2, Figure S3).

**Figure 1:**
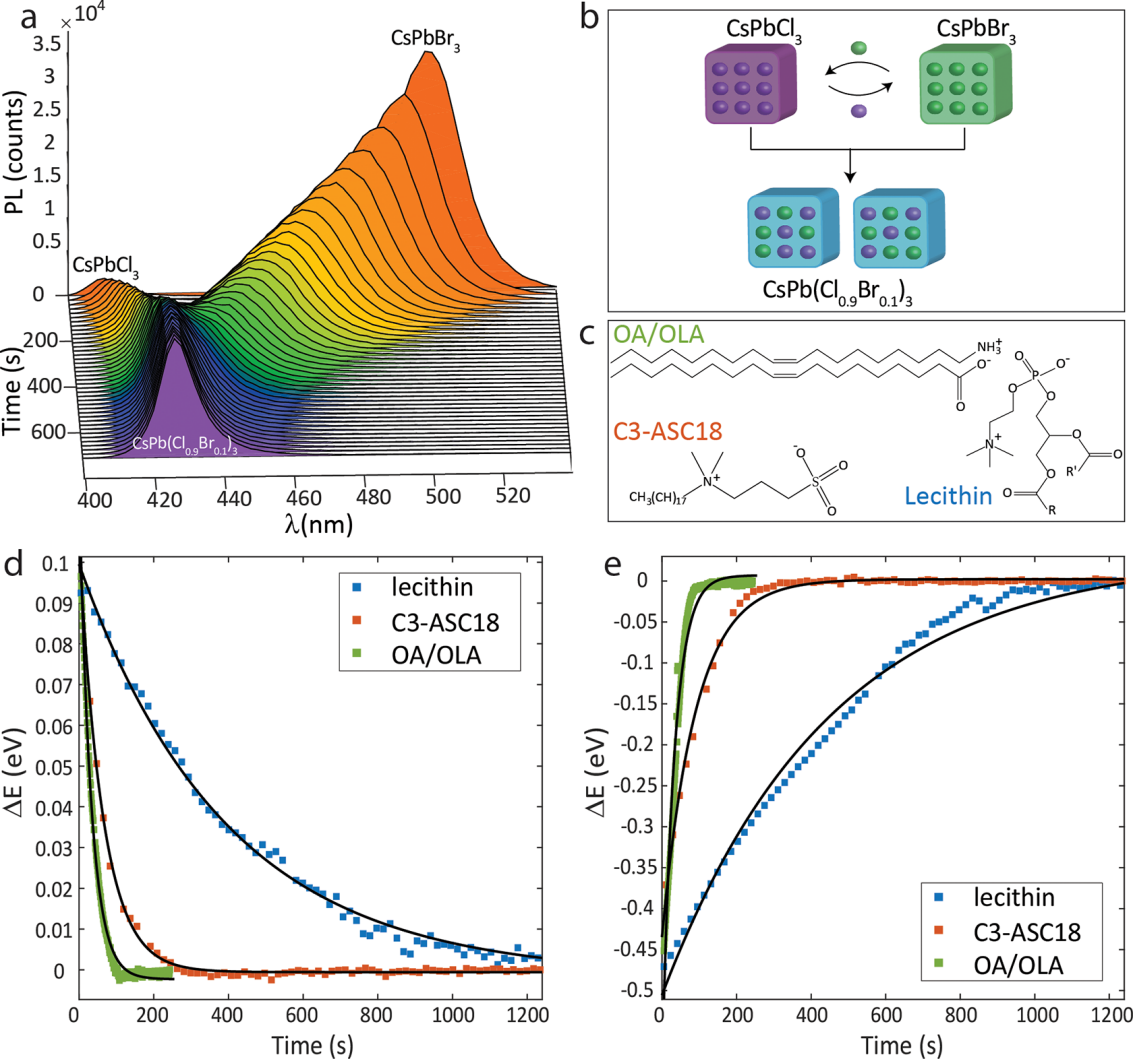
(a) Photoluminescence spectra of the cross-anion exchange reaction kinetics (18 s intervals), of C3-ASC18 capped perovskite NCs. (b) Scheme of the cross-anion exchange reaction. (c) Scheme of the capping ligands. (d) Plot of the energy difference for the PL peak associated with chloride enriched NCs population (high energy peak) at time t relative to its value at long t, for the three different capping ligands (blue squares, lecithin; red squares, C3-ASC18; green squares, OA/OLA), at 25 °C. Solid lines represent the corresponding exponential fittings. (e) Similarly, for the PL peak associated with bromide enriched NCs population (low energy peak).

The spectra were fit to a sum of two Gaussians (Figure S5). The shift in the peak position of each, relative to the equilibrium position at long time, was plotted versus the reaction time. Figure 1d compares the shift of the emission peak versus time of the chloride enriched NCs population (high energy peak) in three systems: the lecithin and C3-ASC18 capped NCs (both are zwitterionic ligands, lecithin is a mixture of molecules with varying R groups),^29^ and the monoionic OA/OLA capped NCs. The corresponding energy difference plot of the bromide enriched NCs population (low energy peak) is presented in Figure 1e. An exponential behavior is seen, consistent with previous reports for the OA/OLA system, in-line with first- or pseudo-first-order reaction kinetics.^25^ The rate constant of the cross-anion exchange reaction was found to be independent of the halide ratio, also consistent with the pseudo-first-order kinetics (SI, section 2). To further establish the kinetic analysis and to solidify the determination of the reaction order of the newly studied zwitterionic coated NCs, the initial-rates method was implemented by studying the reaction kinetics at a range of different initial NCs concentrations. This yielded reaction orders of 1.1 ± 0.3 and 1.3 ± 0.1 for lecithin and C3-ASC18, respectively (SI, section 9, Figure S6).^30^ Thus, continuing with the first-order reaction kinetic analysis, similar rate constants were extracted for both the high and low energy peaks (Table S1), indicating that both reactions proceeded in parallel. To avoid duplicity, we will focus our analysis to the changes in the high energy peak.

The quantitative analysis for the three capping ligands shows that the fastest rate constant for cross-anion exchange is associated with the OA/OLA capped NCs system (0.032 ± 0.001 s^–1^ for the high energy peak, or 0.023 ± 0.005 s^–1^ for 9 nm NCs, see Figure S12), in agreement with previous reports (∼0.035 s^–1^).^20^ This is slowed down significantly for the zwitterionic ligands, decreasing in the case of the C3-ASC18 ligand (0.016 ± 0.001 s^–1^), and falling roughly by an order of magnitude for lecithin (0.003 ± 0.001 s^–1^). The observed results are affected by the size of the NCs, yet the capping ligand is the dominant contributor to the trend (Figure S12). This showcases the capacity of the zwitterionic ligands, especially lecithin, to stabilize the NCs and to maintain their compositional integrity.^29^

To establish the full rate equation and a reaction model, focusing on the better stabilizing zwitterionic ligands, we further determine the ligand’s role. The working hypothesis for the reaction mechanism is that the NC surface ligands shuttle the anions in the cross-exchange reaction. While earlier reports already suggested this,^23,31^ a clear experimental proof is warranted to rule out the possibility of a reaction through a direct collision between the NCs.

To this end, the cross-anion exchange reaction was conducted in a setup comprising a semipermeable membrane allowing for the transfer of ligands but not of the NCs (scheme in Figure 2a, experimental details in SI, section 10). Figure 2b presents the PL spectra from inside and outside the dialysis bag at *t* = 0 and at the conclusion of the measurement, after 7 h. At t = 0 (solid lines) the PL inside/outside correspond to the pure CsPbCl_3_/CsPbBr_3_ NCs peaked at 405/507 nm, respectively. At *t* = 7 h (dashed lines) the PL inside the bag red-shifted to 410 nm, while outside the PL blue-shifted to 459 nm. This showcases that a cross-anion exchange reaction occurred through the membrane, which proves the role of the ligands as halide carriers in this reaction. Compared to the fully equilibrated cross-anion exchange reaction without the presence of the membrane (blue shaded PL peak after 20 min), even after 7 h, equilibrium was not reached in this case, in line with the diffusion barrier imposed by the membrane.

**Figure 2:**
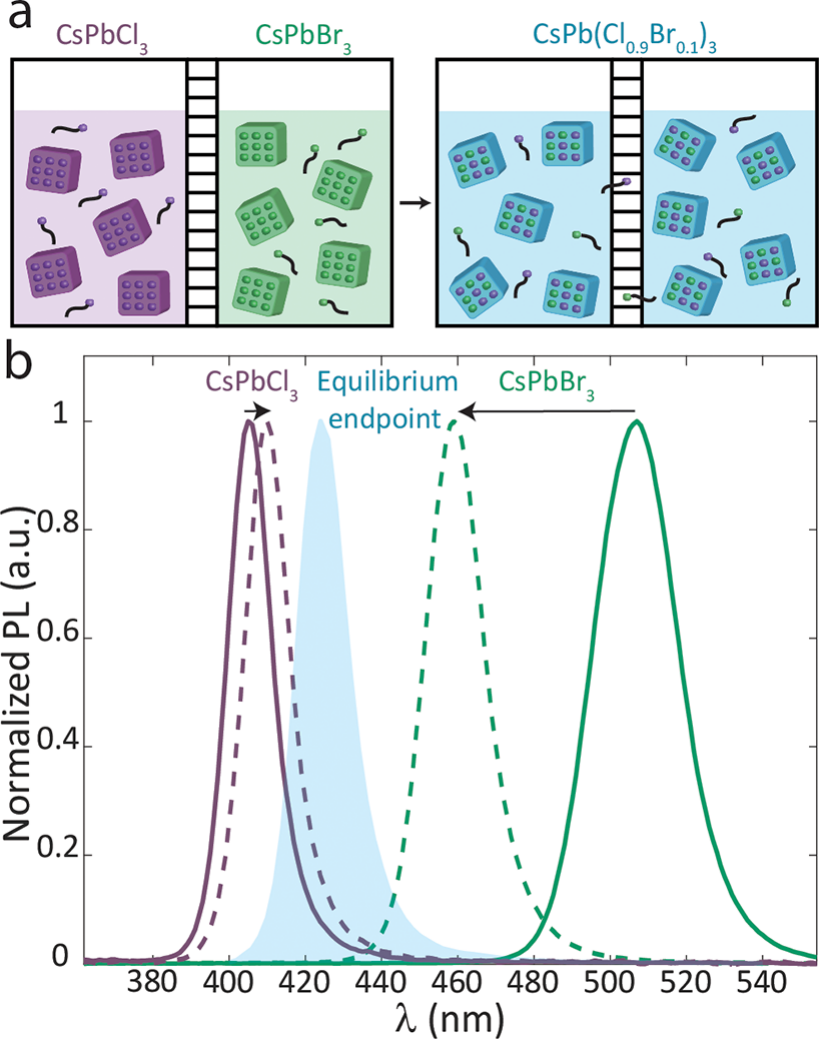
(a) Schematic of the cross-anion exchange reaction through a diffusive membrane (1000 kDa pore size). (b) PL spectra of the CsPbCl_3_ (purple) and CsPbBr_3_ (green) NCs from inside/outside the dialysis bag at time *t* = 0 (solid lines), and at time *t* = 7 h (dashed lines) after the NCs were left to react through the membrane. The blue peak at 425 nm corresponds with a fully equilibrated solution of CsPb(Cl_0.9_Br_0.1_)_3_ NCs, achieved under a no-membrane setup.

Further proof to the direct involvement of ligands in the reaction can be achieved by exchanging NCs with different ligands. The anion-exchange between lecithin capped CsPbCl_3_ and C3-ASC18 capped CsPbBr_3_ yielded a rate constant in the range between those for pure lecithin and pure C3-ASC18 (0.012 ± 0.001 s^–1^, Figure S10a). The same cross-anion exchange reaction with different ligands was performed in a dialysis bag as well. The PL peaks shifted both inside and outside of the dialysis bag, showing that anion-exchange occurs through the membrane also when two types of ligands are involved (Figure S10b). Measuring the Fourier-transform infrared (FTIR) spectrum of the C3-ASC18 capped CsPbBr_3_ outside the dialysis bag after 7 h revealed a C=O stretching peak at 1735 cm^–1^, which is characteristic of lecithin that diffused through the membrane (Figure S10c). We thus directly substantiate the role of ligands in shuttling of the halides in the cross-anion exchange process.

Continuing the kinetic study, the temperature dependence was studied revealing an Arrhenius dependence (Figure 3, Figure S11). The activation energies of the three systems are nearly identical within error, 57 ± 4, 56 ± 2, and 61 ± 6 kJ/mol for the lecithin, C3-ASC18, and OA/OLA capped NCs, respectively, suggesting a similar mechanism and rate-limiting step for all ligands. Comparing two sizes of OA/OLA coated NCs revealed that the activation energy does not depend strongly on the NCs size (Figure S12). All three ligands bind to the surface halides through an ammonium group.^32^ Accordingly, and in line with the establishment of the ligands as halide carriers, we associate the activation energy to the dissociation of the halide-ligand complexes from the NC surface. Nevertheless, the activation energy of anion exchange in OA/OLA capped NCs is slightly higher, consistent with the higher surface coverage of OA/OLA relative to the zwitterionic ligands, as was reported previously.^27^ A denser surface can increase the required energy for ligand desorption due to ligand–ligand interactions.^33^ We note that a previous study for anion exchange between OA/OLA capped CsPbBr_3_ NCs and oleylammonium chloride reported an activation energy of 32 ± 1 kJ/mol.^20^ This result is approximately half of the activation energy for the cross-anion exchange reaction between OA/OLA capped NCs. This demonstrates a difference between the cross-anion exchange reaction and anion exchange with a halide salt. In the case of a cross-anion exchange, mutual ligand dissociation, from the source NC and the target NC, has to occur for a fruitful halide exchange, whereas in the case of anion exchange with halide salt, only one dissociation event is required.

**Figure 3:**
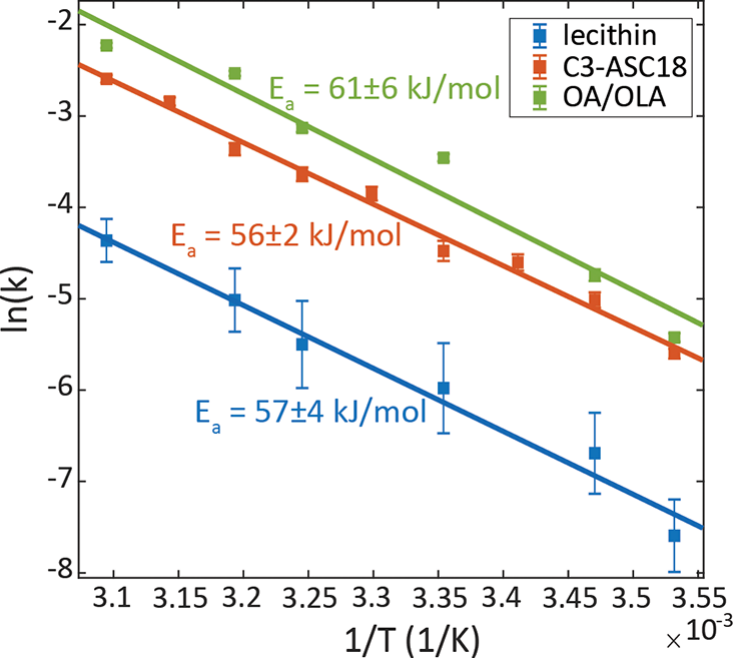
Arrhenius plot for the reaction in the temperature range of 10–50 °C for lecithin (blue), C3-ASC18 (red), and OA/OLA (green) capped NCs.

We next devise a mechanism for the cross-anion exchange reaction. It should reflect a first-order behavior with respect to the halide concentration. However, the influence of the ligand type on the rate, as well as its apparent involvement in the reaction’s mechanism, does not follow an elementary first-order reaction. Instead, a pseudo-first-order rate must be considered. To account for these requirements, we suggest a mechanism that involves the encounter between a halide site on the NC surface and a halide-carrying ligand (SI, section 13). The mechanism is inspired by the “cage effect” for bimolecular reactions in solution,^34,35^ in which two molecules in solution diffuse into a cage of solvent molecules, where they experience multiple encounters and can react with one another or escape out of the cage.

Formulating the mechanism, the net reaction of a cross-anion exchange between CsPbCl_3_ and CsPbBr_3_ NCs with an 8.5:1 ratio of chloride and bromide ions is as follows
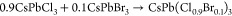
R1

The surface ligands attach and detach from the surface according to their equilibrium constant.^31^ Therefore, a surface site can be either with or without a bound ligand
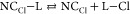
R2.1
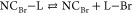
R2.2Where NC_X_ is the relevant halide site on the surface of the NC, and L is the ligand.

Upon mixing of CsPbCl_3_ with CsPbBr_3_ NCs, cross-anion exchange occurs, with the ligands as halide carriers. The exchange takes place when a halide-carrying ligand reaches the surface of the target NC. The ligand can either attach to an under-coordinated Pb site on the surface of the NC directly through the halide or exchange its halide with a bound surface halide.^36^ The PL changes arising from either case are indistinguishable, and we treat both cases as one. After the ligands shuttle the anions to the surface of the NCs, rapid vacancies-mediated diffusion homogenizes the NC composition.^19,37^ Considering that the diffusion length is only a few nanometers, this is not the rate-limiting step in the solution cross-anion exchange reaction. The cross-anion exchange reaction occurs simultaneously on CsPbCl_3_ and CsPbBr_3_ NCs until compositional equilibrium is reached, as the NCs themselves are the halide source for the exchange. According to the results, during the reaction both the high and low energy peaks shift with the same rate constant. Consequently, the mechanism of exchange of pure CsPbCl_3_ and CsPbBr_3_ NCs are similar within the available time resolution. Hence, below, we chose to derive the mechanism of the reaction to start with pure CsPbCl_3_ NCs and to end with mixed halide NCs. This mechanism can be applied to the opposite reaction as well
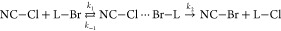
R3Where NC–X is a halide site that can be either bound or unbound to a ligand. NC–Cl···Br–L is an intermediate metastable complex of the reactants within a cage of solvent molecules.

The reaction thus follows the well-established “cage effect” mechanism. The reactants are a chloride site on the surface of the NC and a bromide-carrying ligand. According to the “cage effect”, the molecules diffuse in solution and encounter one another within a cage of solvent molecules. The diffusion into the cage is described by the reaction with rate constant *k*_1_. After reaching the solvent cage, the molecules can react by exchanging their anions (rate constant of *k*_2_), or they can escape the cage (rate constant of *k*_–1_). To simplify the rate equation, a steady-state approximation for the intermediate caged species can be applied, yielding
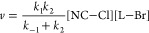
1The rate expression describes a second-order reaction with respect to the chloride sites and the bromide carrying ligands. It becomes a pseudo-first-order reaction in this case because the reaction involves the exchange of anions in different sites, while maintaining a constant concentration of bromide (and chloride) ions. Additionally, the concentration of the ligands in the system is constant. Considering this, the concentration of the ligands that carry bromide ions is effectively constant throughout the entire experiment. Thus, the fixed concentration of ligands with bromide ions can be considered as part of an effective rate constant, *k*_eff_, yielding a pseudo-first-order rate equation
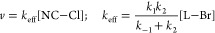
2

To confirm the mechanism, we monitored the reaction rate in the limits of the “cage-effect” mechanism. The kinetics was measured for solutions with varying viscosities (differing in ratios of toluene and octadecene), to test the diffusion dependency (Table S2, Figure S13). Figure 4a presents the ratio between the rate constant in increasing viscosities and the rate constant in toluene alone. The rate of the anion-exchange for both lecithin and C3-ASC18 covered NCs decreased similarly in higher viscosities; however, the absolute rate constants of anion-exchange in lecithin capped NCs are lower than those for C3-ASC18 capped NCs (Figure 4a inset). The lower anion-exchange rate between lecithin capped NCs can be explained by the bulkier structure of lecithin that slows its diffusion. In 90% ODE for the lecithin-capped NCs, the reaction was still not completed even after 24 h, hence we could not approach the limit of full diffusion control. At highly viscous media, the diffusion in and out of the cage is the rate-determining step. The rate constant *k*_–1_ is negligible relative to *k*_2_, and the effective rate of the reaction depends on *k*_1_ alone, agreeing with full diffusion control.
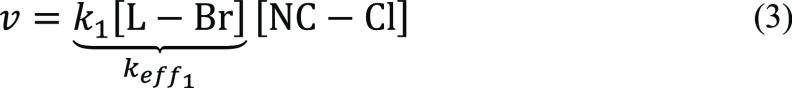
3

**Figure 4:**
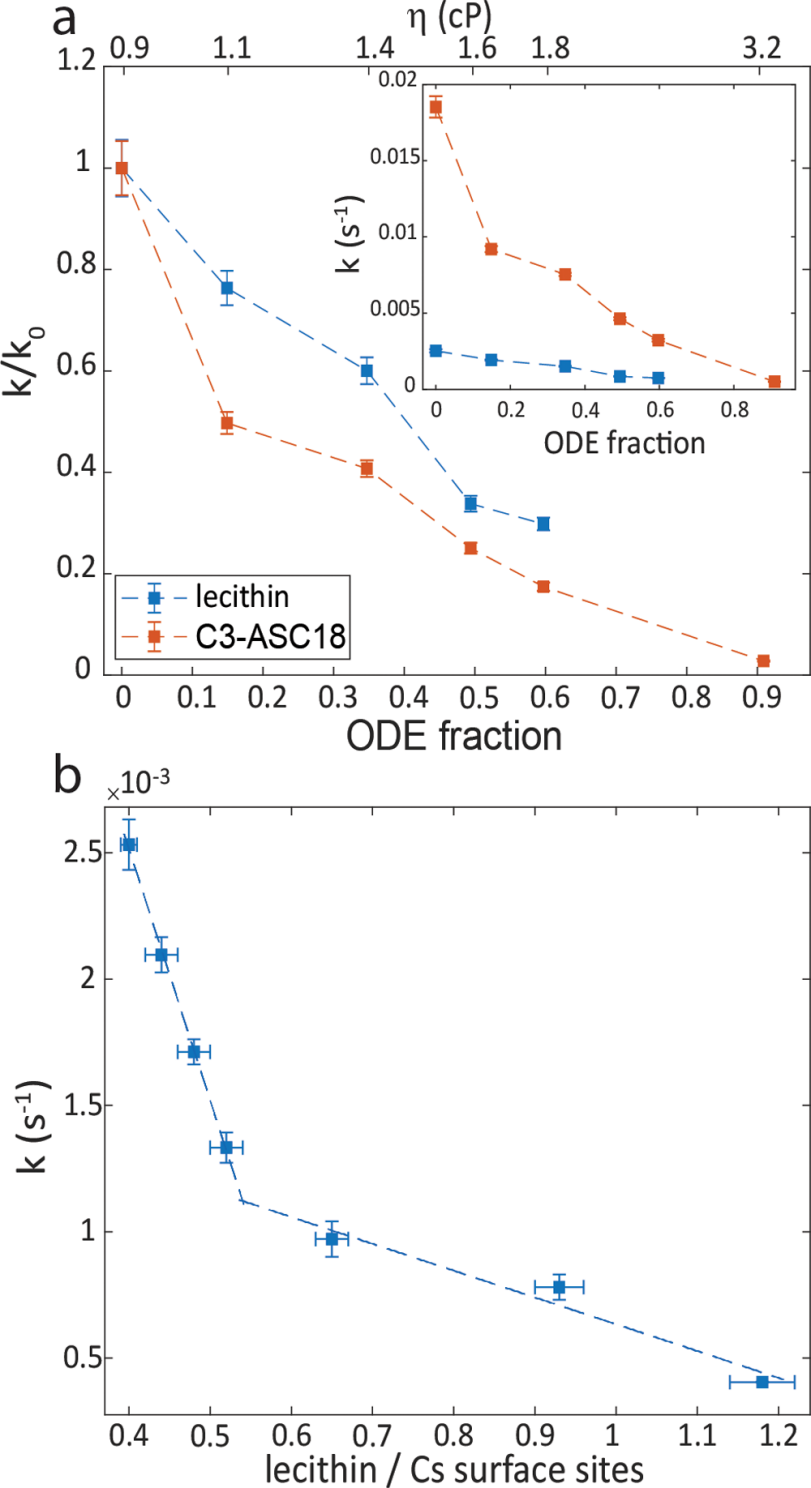
Testing the limits of the “cage effect” mechanism. (a) Viscosity effect on the rate of the cross-anion exchange reaction for lecithin (blue) and C3-ASC18 (red) capped NCs. The varying viscosities are achieved by different ratios of toluene to the more viscous octadecene (ODE). (b) Excess ligand effect on the rate of the anion-exchange reaction in the lecithin capped NCs system. The guidelines emphasize two different trends.

The anion exchange reaction is driven thermodynamically by the entropic gain from mixing of the anions in the lattice, which is a strong factor dictating compositional homogeneity in the NCs. The use of zwitterioinc surface ligands to gain compositional stability indeed effectively slows down the reaction, especially for lecithin. However, according to the proposed mechanism, the reaction rate may be affected by ligands concentration in different ways. On one hand, increasing the ligands concentration may hasten the exchange by providing further means for the cross-anion shuttling. On the other hand, excess ligands can increase the density of the ligand packing, providing increased stability by its passivating effect, hindering the arrival and departure of the ligand-anion complexes to and from the NC surface.

To study these counteracting effects, we examined the dependence of the reaction’s kinetics on excess ligands concentration. These experiments were only feasible with additional lecithin, due to the low solubility of C3-ASC18 in toluene. The initial lecithin surface coverage was determined by thermogravimetric analysis to be 40% relative to the Cs surface sites for both the CsPbCl_3_ and the CsPbBr_3_ NCs (Figure S14a). Cs surface sites were considered because the ammonium group of the bound ligands substitutes Cs atom on the surface of the NCs.^32,36^ Excess ligands at varying concentrations relative to the overall Cs surface sites were added (SI, section 15b). The anion exchange kinetics were studied and analyzed (Figure S14b).

Figure 4b presents the dependence of the rate constant on the ratio of lecithin to Cs surface sites. Increasing the ligand concentration slows down the exchange reaction considerably. The presence of excess ligands enhances the ligand binding and stabilizes the NCs while driving reactions R2.1, R2.2 to shift backward, toward higher surface coverage. We observe two regimes. Up to a ligand:Cs surface sites ratio of ∼0.55, a sharp decrease in the anion exchange rate is observed. Upon ligand addition for a ligand:Cs surface sites ratio higher than 0.6, the rate continues to decrease but with a much weaker dependence on the concentration of the excess ligand, as the stabilizing effect becomes saturated. At 60% surface coverage the surface is practically saturated, as a result of steric and electrostatic effects that prevent 100% surface coverage. Therefore, excess ligands have a positive stabilizing effect but only up to an effective complete ligand coverage.

In summary, a kinetic study of cross-anion exchange in perovskite NCs was presented. The reaction proceeds by the shuttling of anions transported by a ligand-anion complex as proven through its occurrence even in the presence of a membrane barrier impeding the NCs crossing. A “cage effect” mechanism is established, while the observed pseudo first-order dependency on the halide sites’ concentration arises from the presence of an equilibrium concentration of ligand-anion complexes in solution. A comparison of different ligands revealed the significance of surface effects on the reaction, as different surface cappings influenced the stability of the system. Zwitterionic capping ligands provided enhanced passivation, resulting in slower rates of anion exchange. NCs with lecithin as the capping ligand exhibited the highest stability and inhibition of anion exchange due to the bulky structure of lecithin. Although lecithin contains a mixture of carbohydrate tails,^29^ its bulkiness relative to the other studied ligands is mostly attributed to its two long chains, rather than just one. Consequently, lecithin diffuses more slowly in the solution. A more significant effect is surface-related, as it is more difficult for the bulky lecithin to penetrate the ligand layer on the surface. These effects explain the slower rates of cross-anion exchange in lecithin capped NCs. In the design of applications based on perovskite NCs, lecithin capping offers enhanced surface stabilization, and using excess ligands up to a ratio of complete coverage is beneficial. This study sheds light on the mechanism of the cross-anion exchange reaction in perovskite NCs and surface effects. It provides guidelines for slowing down the spontaneous change due to uncontrolled anion exchange with the potential to improve their implementation in various applications.

## Supporting Information Available

Experimental details and methods, a detailed kinetic analysis, and the cross-anion exchange mechanism (PDF)

## References

[ref1] Kovalenko M. V.; Protesescu L.; Bodnarchuk M. I. Properties and Potential Optoelectronic Applications of Lead Halide Perovskite Nanocrystals. Science. 2017, 358, 745–750. 10.1126/science.aam7093. 29123061

[ref2] Akkerman Q. A.; Rainò G.; Kovalenko M. V.; Manna L. Genesis, Challenges and Opportunities for Colloidal Lead Halide Perovskite Nanocrystals. Nat. Mater. 2018, 17 (5), 394–405. 10.1038/s41563-018-0018-4. 29459748

[ref3] Shamsi J.; Urban A. S.; Imran M.; De Trizio L.; Manna L. Metal Halide Perovskite Nanocrystals: Synthesis, Post-Synthesis Modifications, and Their Optical Properties. Chem. Rev. 2019, 119 (5), 3296–3348. 10.1021/acs.chemrev.8b00644. 30758194 PMC6418875

[ref4] Stranks S. D.; Snaith H. J. Metal-Halide Perovskites for Photovoltaic and Light-Emitting Devices. Nature Nanotechnology. 2015, 10, 391–402. 10.1038/nnano.2015.90. 25947963

[ref5] Song J.; Fang T.; Li J.; Xu L.; Zhang F.; Han B.; Shan Q.; Zeng H. Organic–Inorganic Hybrid Passivation Enables Perovskite QLEDs with an EQE of 16.48%. Adv. Mater. 2018, 30 (50), 1805409 10.1002/adma.201805409. 30306653

[ref6] Sadhanala A.; Ahmad S.; Zhao B.; Giesbrecht N.; Pearce P. M.; Deschler F.; Hoye R. L. Z.; Gödel K. C.; Bein T.; Docampo P.; Dutton S. E.; De Volder M. F. L.; Friend R. H. Blue-Green Color Tunable Solution Processable Organolead Chloride-Bromide Mixed Halide Perovskites for Optoelectronic Applications. Nano Lett. 2015, 15 (9), 6095–6101. 10.1021/acs.nanolett.5b02369. 26236949 PMC4762541

[ref7] Su L.; Zhao Z. X.; Li H. Y.; Yuan J.; Wang Z. L.; Cao G. Z.; Zhu G. High-Performance Organolead Halide Perovskite-Based Self-Powered Triboelectric Photodetector. ACS Nano 2015, 9 (11), 11310–11316. 10.1021/acsnano.5b04995. 26469207

[ref8] Wei H.; Fang Y.; Mulligan P.; Chuirazzi W.; Fang H. H.; Wang C.; Ecker B. R.; Gao Y.; Loi M. A.; Cao L.; Huang J. Sensitive X-Ray Detectors Made of Methylammonium Lead Tribromide Perovskite Single Crystals. Nat. Photonics 2016, 10 (5), 333–339. 10.1038/nphoton.2016.41.

[ref9] Nie W.; Tsai H.; Asadpour R.; Blancon J. C.; Neukirch A. J.; Gupta G.; Crochet J. J.; Chhowalla M.; Tretiak S.; Alam M. A.; Wang H. L.; Mohite A. D. High-Efficiency Solution-Processed Perovskite Solar Cells with Millimeter-Scale Grains. Science (80-.) 2015, 347 (6221), 522–525. 10.1126/science.aaa0472. 25635093

[ref10] Kim J. Y.; Lee J.-W.; Jung H. S.; Shin H.; Park N.-G. High-Efficiency Perovskite Solar Cells. Chem. Rev. 2020, 120 (15), 7867–7918. 10.1021/acs.chemrev.0c00107. 32786671

[ref11] Lin J.; Lai M.; Dou L.; Kley C. S.; Chen H.; Peng F.; Sun J.; Lu D.; Hawks S. A.; Xie C.; Cui F.; Alivisatos A. P.; Limmer D. T.; Yang P. Thermochromic Halide Perovskite Solar Cells. Nat. Mater. 2018, 17 (3), 261–267. 10.1038/s41563-017-0006-0. 29358645

[ref12] Pellet N.; Teuscher J.; Maier J.; Grätzel M. Transforming Hybrid Organic Inorganic Perovskites by Rapid Halide Exchange. Chem. Mater. 2015, 27 (6), 2181–2188. 10.1021/acs.chemmater.5b00281.

[ref13] Protesescu L.; Yakunin S.; Bodnarchuk M. I.; Krieg F.; Caputo R.; Hendon C. H.; Yang R. X.; Walsh A.; Kovalenko M. V. Nanocrystals of Cesium Lead Halide Perovskites (CsPbX_3_, X = Cl, Br, and I): Novel Optoelectronic Materials Showing Bright Emission with Wide Color Gamut. Nano Lett. 2015, 15 (6), 3692–3696. 10.1021/nl5048779. 25633588 PMC4462997

[ref14] Nedelcu G.; Protesescu L.; Yakunin S.; Bodnarchuk M. I.; Grotevent M. J.; Kovalenko M. V. Fast Anion-Exchange in Highly Luminescent Nanocrystals of Cesium Lead Halide Perovskites (CsPbX_3_, X = Cl, Br, I). Nano Lett. 2015, 15 (8), 5635–5640. 10.1021/acs.nanolett.5b02404. 26207728 PMC4538456

[ref15] Akkerman Q. A.; D’Innocenzo V.; Accornero S.; Scarpellini A.; Petrozza A.; Prato M.; Manna L. Tuning the Optical Properties of Cesium Lead Halide Perovskite Nanocrystals by Anion Exchange Reactions. J. Am. Chem. Soc. 2015, 137 (32), 10276–10281. 10.1021/jacs.5b05602. 26214734 PMC4543997

[ref17] Zhang Y.; Lu D.; Gao M.; Lai M.; Lin J.; Lei T.; Lin Z.; Quan L. N.; Yang P. Quantitative Imaging of Anion Exchange Kinetics in Halide Perovskites. Proc. Natl. Acad. Sci. U. S. A. 2019, 116 (26), 12648–12653. 10.1073/pnas.1903448116. 31189607 PMC6601281

[ref18] Pan D.; Fu Y.; Chen J.; Czech K. J.; Wright J. C.; Jin S. Visualization and Studies of Ion-Diffusion Kinetics in Cesium Lead Bromide Perovskite Nanowires. Nano Lett. 2018, 18 (3), 1807–1813. 10.1021/acs.nanolett.7b05023. 29397750

[ref19] Lai M.; Obliger A.; Lu D.; Kley C. S.; Bischak C. G.; Kong Q.; Lei T.; Dou L.; Ginsberg N. S.; Limmer D. T.; Yang P. Intrinsic Anion Diffusivity in Lead Halide Perovskites Is Facilitated by a Soft Lattice. Proc. Natl. Acad. Sci. U. S. A. 2018, 115 (47), 11929–11934. 10.1073/pnas.1812718115. 30397127 PMC6255190

[ref20] Koscher B. A.; Bronstein N. D.; Olshansky J. H.; Bekenstein Y.; Alivisatos A. P. Surface- vs Diffusion-Limited Mechanisms of Anion Exchange in CsPbBr_3_ Nanocrystal Cubes Revealed through Kinetic Studies. J. Am. Chem. Soc. 2016, 138 (37), 12065–12068. 10.1021/jacs.6b08178. 27606934

[ref21] Li M.; Zhang X.; Wang P.; Yang P. Metastable γ-CsPbI_3_ Perovskite Nanocrystals Created Using Aged Orthorhombic CsPbBr_3_. J. Phys. Chem. C 2021, 125 (13), 7109–7118. 10.1021/acs.jpcc.0c09672.

[ref22] Abdel-Latif K.; Epps R. W.; Kerr C. B.; Papa C. M.; Castellano F. N.; Abolhasani M. Facile Room-Temperature Anion Exchange Reactions of Inorganic Perovskite Quantum Dots Enabled by a Modular Microfluidic Platform. Adv. Funct. Mater. 2019, 29 (23), 1900712 10.1002/adfm.201900712.

[ref23] Haque A.; Chonamada T. D.; Dey A. B.; Santra P. K. Insights into the Interparticle Mixing of CsPbBr_3_ and CsPbI_3_ nanocubes: Halide Ion Migration and Kinetics. Nanoscale 2020, 12 (40), 20840–20848. 10.1039/D0NR05771A. 33043328

[ref24] Kamat P. V.; Kuno M. Halide Ion Migration in Perovskite Nanocrystals and Nanostructures. Acc. Chem. Res. 2021, 54 (3), 520–531. 10.1021/acs.accounts.0c00749. 33475338

[ref25] Ravi V. K.; Scheidt R. A.; Nag A.; Kuno M.; Kamat P. V. To Exchange or Not to Exchange. Suppressing Anion Exchange in Cesium Lead Halide Perovskites with PbSO_4_-Oleate Capping. ACS Energy Lett. 2018, 3 (4), 1049–1055. 10.1021/acsenergylett.8b00380.

[ref26] Loiudice A.; Strach M.; Saris S.; Chernyshov D.; Buonsanti R. Universal Oxide Shell Growth Enables in Situ Structural Studies of Perovskite Nanocrystals during the Anion Exchange Reaction. J. Am. Chem. Soc. 2019, 141 (20), 8254–8263. 10.1021/jacs.9b02061. 31045360

[ref27] Krieg F.; Ochsenbein S. T.; Yakunin S.; Ten Brinck S.; Aellen P.; Süess A.; Clerc B.; Guggisberg D.; Nazarenko O.; Shynkarenko Y.; Kumar S.; Shih C. J.; Infante I.; Kovalenko M. V. Colloidal CsPbX_3_ (X = Cl, Br, I) Nanocrystals 2.0: Zwitterionic Capping Ligands for Improved Durability and Stability. ACS Energy Lett. 2018, 3 (3), 641–646. 10.1021/acsenergylett.8b00035. 29552638 PMC5848145

[ref28] Pan J.; Shang Y.; Yin J.; De Bastiani M.; Peng W.; Dursun I.; Sinatra L.; El-Zohry A. M.; Hedhili M. N.; Emwas A. H.; Mohammed O. F.; Ning Z.; Bakr O. M. Bidentate Ligand-Passivated CsPbI_3_ Perovskite Nanocrystals for Stable Near-Unity Photoluminescence Quantum Yield and Efficient Red Light-Emitting Diodes. J. Am. Chem. Soc. 2018, 140 (2), 562–565. 10.1021/jacs.7b10647. 29249159

[ref29] Krieg F.; Ong Q. K.; Burian M.; Rainò G.; Naumenko D.; Amenitsch H.; Süess A.; Grotevent M. J.; Krumeich F.; Bodnarchuk M. I.; Shorubalko I.; Stellacci F.; Kovalenko M. V. Stable Ultraconcentrated and Ultradilute Colloids of CsPbX_3_ (X = Cl, Br) Nanocrystals Using Natural Lecithin as a Capping Ligand. J. Am. Chem. Soc. 2019, 141 (50), 19839–19849. 10.1021/jacs.9b09969. 31763836 PMC6923794

[ref30] Mortimer M.; Taylor P.; Smart L. E.; Clark G. The Open University. Chemical Kinetics and Mechanism; Royal Society of Chemistry, Cambridge, UK, 2002; pp 60–62.

[ref31] De Roo J.; Ibáñez M.; Geiregat P.; Nedelcu G.; Walravens W.; Maes J.; Martins J. C.; Van Driessche I.; Kovalenko M. V.; Hens Z. Highly Dynamic Ligand Binding and Light Absorption Coefficient of Cesium Lead Bromide Perovskite Nanocrystals. ACS Nano 2016, 10 (2), 2071–2081. 10.1021/acsnano.5b06295. 26786064

[ref32] Ravi V. K.; Santra P. K.; Joshi N.; Chugh J.; Singh S. K.; Rensmo H.; Ghosh P.; Nag A. Origin of the Substitution Mechanism for the Binding of Organic Ligands on the Surface of CsPbBr_3_ Perovskite Nanocubes. J. Phys. Chem. Lett. 2017, 8 (20), 4988–4994. 10.1021/acs.jpclett.7b02192. 28937765

[ref33] Elimelech O.; Aviv O.; Oded M.; Banin U. A Tale of Tails: Thermodynamics of CdSe Nanocrystal Surface Ligand Exchange. Nano Lett. 2020, 20 (9), 6396–6403. 10.1021/acs.nanolett.0c01913. 32787157

[ref34] Franck J.; Rabinowitsch E. Some Remarks about Free Radicals and the Photochemistry of Solutions. Trans. Faraday Soc. 1934, 30, 120–130. 10.1039/tf9343000120.

[ref35] Noyes R. M. The Recombination of Iodine Atoms in Solution. J. Chem. Phys. 1950, 18 (8), 999–1002. 10.1063/1.1747898.

[ref36] Nenon D. P.; Pressler K.; Kang J.; Koscher B. A.; Olshansky J. H.; Osowiecki W. T.; Koc M. A.; Wang L. W.; Alivisatos A. P. Design Principles for Trap-Free CsPbX_3_ Nanocrystals: Enumerating and Eliminating Surface Halide Vacancies with Softer Lewis Bases. J. Am. Chem. Soc. 2018, 140 (50), 17760–17772. 10.1021/jacs.8b11035. 30501174

[ref37] Steele J. A.; Lai M.; Zhang Y.; Lin Z.; Hofkens J.; Roeffaers M. B. J.; Yang P. Phase Transitions and Anion Exchange in All-Inorganic Halide Perovskites. Accounts Mater. Res. 2020, 1 (1), 3–15. 10.1021/accountsmr.0c00009.

