# Supplementary material for: Ligands Mediate Anion Exchange between Colloidal Lead-Halide Perovskite Nanocrystals
Source: Nano Lett. 2022 May 23;22(11):4340–6. doi: 10.1021/acs.nanolett.2c00611 (PMC9185745; doi:10.1021/acs.nanolett.2c00611)
Supplement: Supplementary file 1 — nl2c00611_si_001.pdf [file nl2c00611_si_001.pdf]

Supporting Information

Ligands mediate anion exchange between colloidal lead-halide perovskite nanocrystals

Einav Scharf[1], Franziska Krieg[2,3,#], Orian Elimelech[1], Meirav Oded[1], Adar Levi[1], Dmitry N. Dirin[2,3], Maksym V. Kovalenko[2,3], and Uri Banin[1,]*

[1] The Institute of Chemistry and the Center for Nanoscience and Nanotechnology, The Hebrew University of Jerusalem, Jerusalem 91904, Israel

[2] Institute of Inorganic Chemistry, Department of Chemistry and Applied Biosciences, ETH, Zürich, Vladimir Prelog Weg 1, CH-8093, Zürich, Switzerland

[3] Laboratory for Thin Films and Photovoltaics, Empa – Swiss Federal Laboratories for Materials Science and Technology, Ueberlandstrasse 129, CH-8600, Dübendorf, Switzerland

Present address: #F.K: Avantama AG, Stäfa, CH8712, Switzerland

Corresponding Author

* E-mail: Uri.Banin@mail.huji.ac.il

Contents

Methods 3

1. Synthesis and characterization 3

2. Cl:Br ratio 10

3. Extinction coefficient 12

4. Halide number in NC and Cs surface sites calculations 13

5. Sample preparation for optical kinetic experiments 14

6. Photoluminescence measurements 14

7. Anion exchange product 16

9. Initial rates method 18

10. Diffusion through a semipermeable membrane 20

11. Temperature effect 24

12. Size dependency 25

13. Mechanism 26

14. Viscosity measurements 27

15. Surface coverage 28

Methods

Structural characterization was done by Transmission Electron Microscope (TEM) Tecnai G^2 Spirit TWIN T12 (ThermoFisher); Aberration Probe-Corrected Scanning Transmission Electron Microscope Themis Z G3 (ThermoFisher); Extra-High Resolution Scanning Electron Microscope (SEM) Magellan 400L (ThermoFisher) for energy-dispersive X-ray spectroscopy (EDS); and by powder X-ray diffraction (XRD). Absorbance measurements were performed with JASCO V-770 UV-VIS-NIR spectrophotometer. Photoluminescence measurements were performed using FLSP920 Fluorescence spectrometer (Edinburgh Instruments), and USB400-UV-VIS-ES (Ocean Insight). Dialysis experiments were performed with Biotech cellulose ester tubing (Spectra/Por®). Viscosity measurements were performed with Brookfield DV-II viscometer. Attenuated total reflection Fourier transform infrared (ATR-FTIR) measurements were performed using a Nicolet iS50 FT-IR spectrometer with Smart iTX accessory (Thermo Scientific). TGA measurements were conducted using TGA-5500 (TA instruments).

1. Synthesis and characterization
    a. Chemicals:

    The following reagents were purchased and used as received:

    Cs_2CO_3, was purchased from Fluorochem, soy-lecithin (>97%, biochemistry grade) from Roth, potassium permanganate from Fluka, $PbBr_2$ (99.99% metal base) from ABCR, lead acetate trihydrate (99.99%), bromine (99.9%), HCl (fuming), 1-octadecene (ODE, technical grade), 3-(N,N-dimethyloctadecylammonio)propanesulfonate (>99% , C3-ASC18), $PbCl_2$ and oleic acid (90%, OA) from Sigma Aldrich/Merck, toluene (for synthesis),

acetone (HPLC grade) and Ethylacetate (HPLC grade) from Fischer and trioctylphosphine (>97%, TOP), and oleylamine (>95%, OLA) from STREM.

b. Synthesis procedures:

Nanocrystals were synthesized according to previously published procedures.[1–3]

i. Cs-oleate 0.4 M in ODE:

Cesium carbonate (1.628 g, 5 mmol), oleic acid (5 mL, 16 mmol) and 1-octadecene (20 mL) were evacuated at 25-120 °C until the completion of gas evolution.

ii. Pb-oleate 0.5 M in ODE:

Lead (II) acetate trihydrate (4.607g, 12 mmol), oleic acid (7.6 mL, 24 mmol) and 1-octadecene (16.4 mL) were mixed in a three-necked flask and evacuated at 25-120 °C until the complete evaporation of acetic acid and water.

iii. TOP-Br_2 0.5 M in toluene:

TOP (6mL, 13 mmol) and bromine (0.6 mL, 11.5 mmol) were mixed under inert atmosphere. Once the reaction was complete and cooled to room temperature, the TOP-Br_2 was dissolved in toluene (18.7 mL).

iv. TOP-Cl_2 0.5 M in toluene:

TOP (30 mL, 0.067 mol) was reacted with Cl_2 gas (ca. 0.34 mol, *i.e.* large excess) at 0 °C. Chlorine gas was produced in-situ by the reaction of concentrated HCl (85 mL, 0.6725 mol) and potassium permanganate (106.27 g) and, after being cleaned from residual HCl by water, was

transferred to the reaction vessel by argon gas flow. The resulting transparent product was diluted with toluene (93 mL).

v. $CsPbBr_3$ nanocrystals with OA/OLA as a ligand:

$CsPbBr_3$ NCs were synthesized by dissolving $PbBr_2$ (0.69g, 1.8 mmol) with oleic acid (OA, 5 mL) and oleylamine (OLA, 5 mL) in ODE (50 mL). The mixture was degassed under vacuum at 120°C for 1h and subsequently heated to 180°C under Argon atmosphere. Once the reaction temperature was reached Cs-Oleate was injected (1.25 mL, 0.5 mmol). The reaction was cooled with an ice water batch immediately after injection. The crude solution was centrifuged at 29500g (g is the earth gravity) for 10 minutes and the precipitate was dispersed in 10 mL of toluene and centrifuged again at 29500g (g is the earth gravity) for 10 minutes to precipitate the fraction of NCs that are not colloidally stable.

vi. $CsPbCl_3$ nanocrystals with OA/OLA as a ligand:

$CsPbCl_3$ NCs were synthesized by dissolving $PbCl_2$ (0.525g, 1.8 mmol) with oleic acid (OA, 5 mL), trioctyl phosphine (TOP, 10 mL) and oleylamine (OLA, 5 mL) in ODE (50 mL). The mixture was degassed under vacuum at 120°C for 1h and subsequently heated to 180°C under Argon atmosphere. Once the reaction temperature was reached Cs-Oleate was injected (1.25 mL, 0.5 mmol). The reaction was cooled with an ice water batch immediately after injection. The crude solution was centrifuged at 29500g (g is the earth gravity) for 10 minutes and the precipitate was dispersed in 10 mL of toluene and

centrifuged again at 29500g (g is the earth gravity) for 10 minutes to precipitate the fraction of NCs that are not colloidally stable.

vii. $CsPbBr_3$ nanocrystals with C3-ASC18 as a ligand:

$CsPbBr_3$ NCs were synthesized by dissolving Cs-oleate (4 mL, 1.6 mmol), Pb-oleate (5 mL, 2.5 mmol) and C3-ASC18 (0.215 g, *ca.* 0.512 mmol) in ODE (5 mL) and heating the mixture under vacuum to 150 °C, whereupon the atmosphere was changed to argon and TOP-Br_2 in toluene (5 mL, 5 mmol of Br) was injected. The reaction was cooled immediately by an ice bath. The crude solution was precipitated by the addition of 2 volumetric equivalents of acetone, followed by the centrifugation at 29500g (g is the earth gravity) for 10 minutes. The precipitated fraction was dispersed in 10 mL of toluene and then washed three more times. Each time the solution was mixed with two volumetric equivalents of acetone and centrifuged at 29500 g for 1 minute, and subsequently dispersed in the progressively smaller amounts of the solvent (5mL for the second cycle, 2.5 mL for the third cycle). After the last precipitation, NCs were dispersed in 2 mL of toluene and centrifuged at 29500 g for 1 minute to remove any non-dispersed residue.

viii. $CsPbCl_3$ nanocrystals with C3-ASC18 as a ligand:

$CsPbCl_3$ NCs were synthesized by dissolving Cs-oleate (4 mL, 1.6 mmol), Pb-oleate (5 mL, 2.5 mmol) and C3-ASC18 (0.215 g, *ca.* 0.512 mmol) in ODE (5 mL) and heating the mixture under vacuum to 130 °C, whereupon the atmosphere was changed to argon and TOP-Cl_2 in toluene (5 mL, 5 mmol of Br) was injected. The reaction was cooled

immediately by an ice bath. The crude solution was precipitated by the addition of 2 volumetric equivalents of acetone, followed by the centrifugation at 29500g (g is the earth gravity) for 10 minutes. The precipitated fraction was dispersed in 10 mL of toluene and then washed three more times. Each time the solution was mixed with two volumetric equivalents of acetone and centrifuged at 29500 g for 1 minute, and subsequently dispersed in the progressively smaller amounts of the solvent (5mL for the second cycle, 2.5 mL for the third cycle). After the last precipitation, NCs were dispersed in 2 mL of toluene and centrifuged at 29500 g for 1 minute to remove any non-dispersed residue.

ix. $CsPbBr_3$ nanocrystals with lecithin as a ligand:

$CsPbBr_3$ NCs were synthesized by dissolving Cs-oleate (4 mL, 1.6 mmol), Pb-oleate (5 mL, 2.5 mmol) and lecithin (0.324 g, *ca.* 0.45 mmol) in ODE (10 mL) and heating the mixture under vacuum to 130 °C, whereupon the atmosphere was changed to argon and TOP-Br_2 in toluene (5 mL, 5 mmol of Br) was injected. The reaction was cooled immediately by an ice bath. The crude solution was precipitated by the addition of 2 volumetric equivalents of acetone, followed by the centrifugation at 29500g (g is the earth gravity) for 10 minutes. The precipitated fraction was dispersed in 10 mL of toluene and then washed three more times. Each time the solution was mixed with two volumetric equivalents of acetone and centrifuged at 29500 g for 1 minute, and subsequently dispersed in the progressively smaller amounts of the solvent (5mL for the second cycle, 2.5 mL for the third

cycle). After the last precipitation, NCs were dispersed in 2 mL of toluene and centrifuged at 29500 g for 1 minute to remove any non-dispersed residue.

x. <u>$CsPbCl_3$ nanocrystals with lecithin as a ligand:</u>

$CsPbCl_3$ NCs were synthesized by dissolving Cs-oleate (4 mL, 1.6 mmol), Pb-oleate (5 mL, 2.5 mmol) and lecithin (0.65 g, *ca.* 0.9 mmol) in ODE (5 mL) and heating the mixture under vacuum to 130 °C, whereupon the atmosphere was changed to argon and TOP-Cl_2 in toluene (5 mL, 5 mmol of Br) was injected. The reaction was cooled immediately by an ice bath. The crude solution was precipitated by the addition of 2 volumetric equivalents of acetone, followed by the centrifugation at 29500g (g is the earth gravity) for 10 minutes. The precipitated fraction was dispersed in 10 mL of toluene and then washed three more times. Each time the solution was mixed with two volumetric equivalents of acetone and centrifuged at 29500 g for 1 minute, and subsequently dispersed in the progressively smaller amounts of the solvent (5mL for the second cycle, 2.5 mL for the third cycle). After the last precipitation, NCs were dispersed in 2 mL of toluene and centrifuged at 29500 g for 1 minute to remove any non-dispersed residue.

Sizes of the nanocrystals (NCs):

OA/OLA:

- $CsPbCl_3$ – 6±1 nm (62 NCs), $CsPbBr_3$ – 7±1 (61 NCs) – kinetic experiments.
- $CsPbCl_3$ – 8.7±0.9 nm (60 NCs), $CsPbBr_3$ – 9.0±0.8 (60 NCs) – size dependency.

C3-ASC18:

- $CsPbCl_3$ – 10±1 nm (61 NCs), $CsPbBr_3$ – 9±1 (61 NCs) – kinetic experiments.
- $CsPbCl_3$ – 10±1 nm (60 NCs), $CsPbBr_3$ – 10±2 (60 NCs) – electron microscopy characterization and diffusion through a semipermeable membrane.

Lecithin: $CsPbCl_3$ – 9±1 nm (60 NCs), $CsPbBr_3$ – 9±1 (61 NCs)

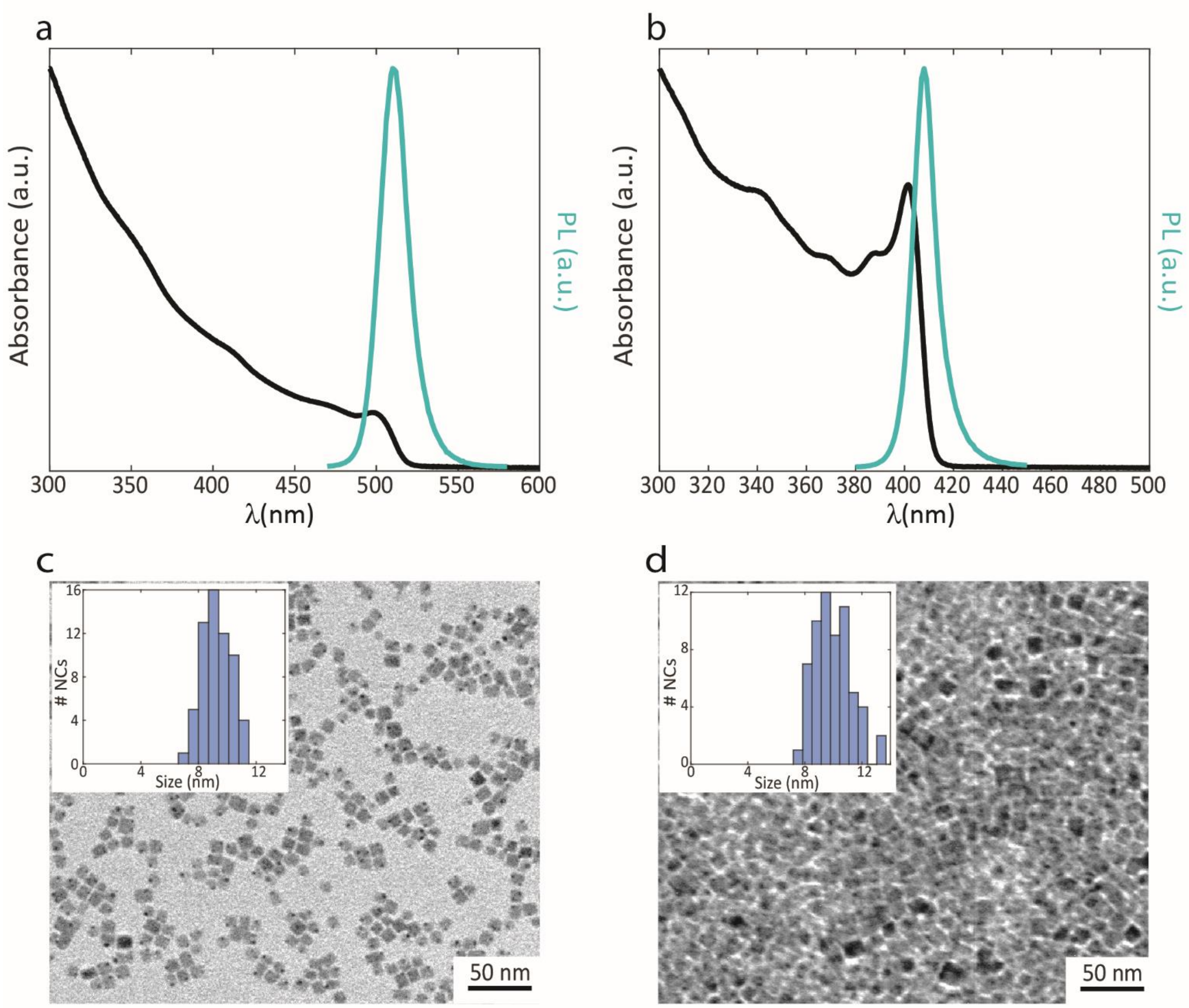

Figure S1. Absorbance and photoluminescence spectra of (a) C3-ASC18 capped $CsPbBr_3$ NCs and (b) C3-ASC18 capped $CsPbCl_3$ NCs. TEM images and size distribution histograms of (c) C3-ASC18 capped $CsPbBr_3$ NCs and (d) C3-ASC18 capped $CsPbCl_3$ NCs.

2. Cl:Br ratio

In order to determine the suitable halides ratio for the cross-anion exchange experiments, focusing in the lecithin capped NCs as a model system, we performed a kinetic cross-anion exchange study with different Cl:Br ratios of 1:3, 3:2 and 8.5:1 (the last reported in the manuscript). The high energy peak reduces to nearly

background level for a Cl:Br ratio of 1:3 and thus we extracted the rate constant of the energy shift in the low energy peak over time (see section 8 for the analysis details). The extracted rate constant of anion exchange in all three ratios was 0.002±0.001 s^{-1} at 25 °C. This independence on the initial concentration ratio is fully consistent with the pseudo first-order kinetics. Considering this and the significantly lower PL quantum yield of $CsPbCl_3$ relative to $CsPbBr_3$ we chose to use a ratio of Cl:Br=8.5:1, in which we were able to detect and reliably analyze also the high energy peak with chloride enriched NCs.

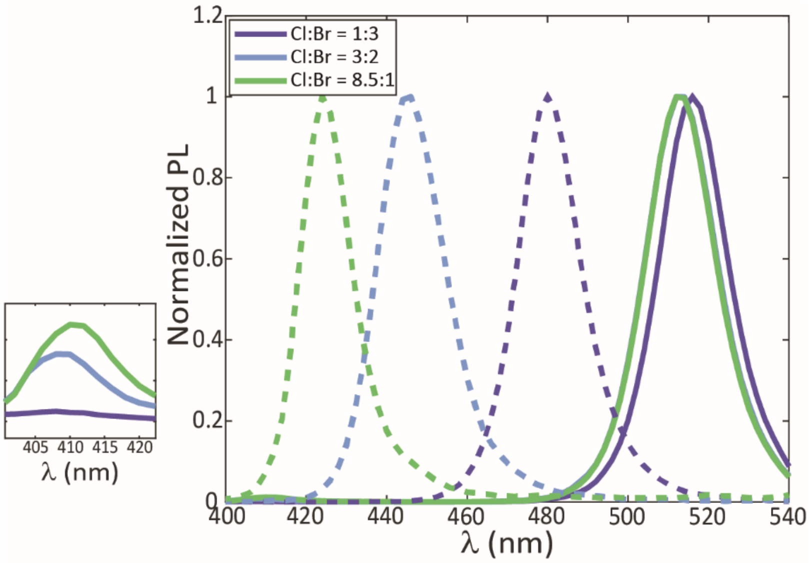

Figure S2. PL spectra at t=5 s after mixing lecithin capped $CsPbCl_3$ NCs with $CsPbBr_3$ NCs with different Cl:Br ratios (solid lines – the 1:3 and 3:2 low energy peaks overlap at this short time). The dashed lines are the respective PL spectra at the end of the anion exchange reactions. The zoom-in shows the much lower emission from the high energy chloride rich NCs PL peaks.

The final Cl:Br ratio in the product NCs was characterized by powder X-ray diffraction (XRD), and by scanning electron microscope-energy-dispersive X-ray spectroscopy (SEM-EDS). According to the XRD peak positions, using Vegard's law,[4] the extracted ratio of chloride anions to bromide anions is 9:1. The SEM-EDS result is 8:1. Therefore, the Cl:Br ratio in the product NCs is 8.5(±0.5):1, and the empirical formula used for the anion-exchange product is $CsPb(Cl_{0.9}Br_{0.1})_3$.

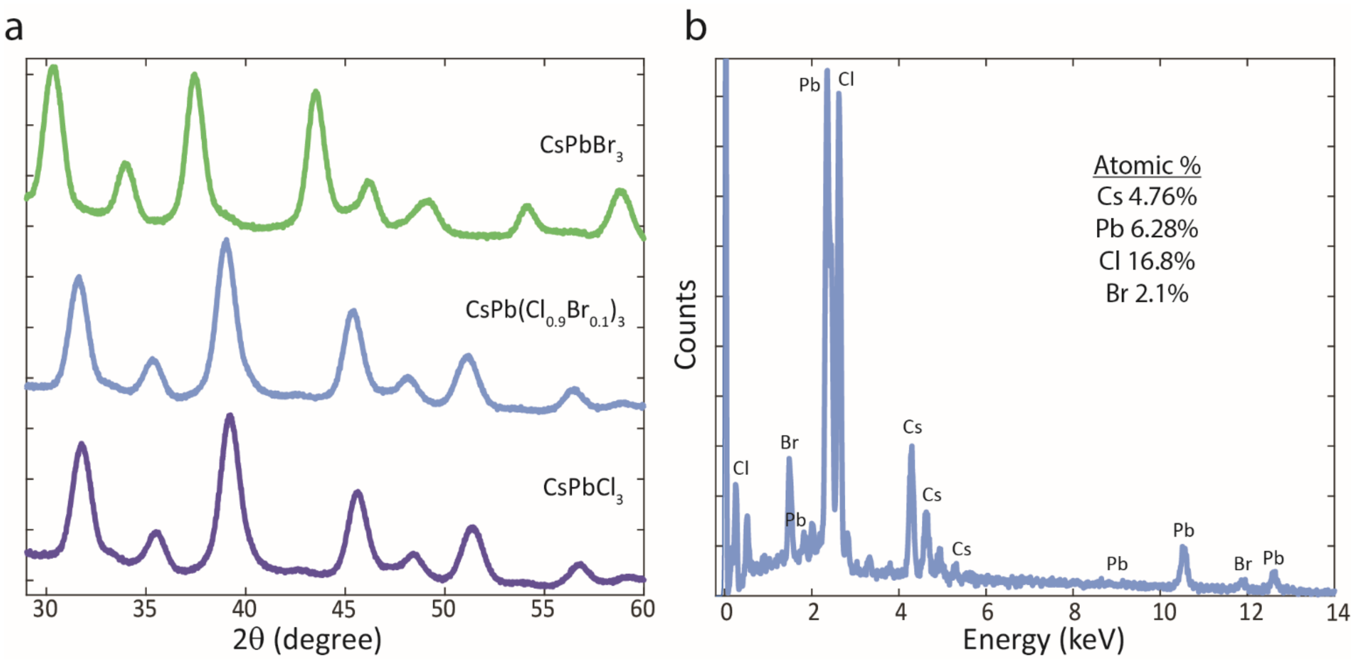

Figure S3. (a) Powder X-ray diffraction patterns of $CsPbBr_3$ (top), $CsPbCl_3$ (bottom), and the product of cross-anion exchange with the empirical formula of $CsPb(Cl_{0.9}Br_{0.1})_3$ according to Vegard's law (middle). (b) SEM-EDS measurement of the product of cross-anion exchange, which provides a ratio of 8:1 between chloride ions and bromide ions. The inset indicates the elemental percentage.

3. <u>Extinction coefficient</u>

Nanocrystal concentrations in solution were determined using their respective extinction coefficients. For $CsPbBr_3$ the published value was used.[5]

The extinction coefficient for $CsPbCl_3$ NCs was estimated using a combination of absorption spectroscopy and TGA. The absorption spectrum was recorded. Then, the same amount was used in order to determine the mass of the inorganic content by TGA measurements.

The absorption spectrum is very variable with size differences due to the large band gap energy and interference of organic materials at energies sufficiently remote of the excitonic transition. The received extinction coefficient is hence a rough estimation and should not be generalized to different NC sizes.

4. <u>Halide number in NC and Cs surface sites calculations</u>

   a. <u>Number of halide ions in a NC:</u>

      The calculation was done under the assumption that the NCs are cubic. The edge of the cube r was found using TEM statistics. The number of $CsPbX_3$ units in a NC:

      Equation S1. $$N = N_{AV} n = N_{AV} \frac{m}{Mw} = N_{AV} \frac{\rho \cdot r^3}{Mw}$$

      Where ρ is the bulk density of $CsPbX_3$.

      The number of halides is three times N.

   b. <u>Cs surface sites:</u>

      The number of Cs surface sites is the ratio between the surface area of the NC and the area of one face of the unit cell. For simplicity the lattice parameters of the cubic bulk phases were used.[6] The lead bromide bond lengths do not vary much between the different phases such that this is a sufficient approximation for our purposes.

      Equation S2. $$N_{Cs\ surface\ sites} = \frac{6 \cdot r^2}{a^2}$$

5. Sample preparation for optical kinetic experiments

The kinetic experiments were conducted in a quartz cuvette containing 2 ml of $CsPbCl_3$ in toluene with O.D. of 1.05.

The kinetic experiment started by swiftly injecting 15 µl of $CsPbBr_3$ in toluene, which contained 0.3 µmol of bromide ions. Accordingly, the ratio between chloride and bromide ions was 8.5:1.

6. Photoluminescence measurements

The PL was measured in the FLSP920 fluorimeter spectrometer. The NCs were excited at 300 nm using Xe-900 lamp (Edinburgh Instruments). The spectra were measured continuously in a range of 400-540 nm with a 2 nm step with high speed photomultiplier tube R7400U (Hamamatsu) detector. The gap between the measurements was 18 seconds.

The C3-ASC18 and OA/OLA measurements were faster and required higher temporal resolution. In this case an additional setup for light collection was used. Following the NCs excitation, as mentioned before, the light was collected using an optical fiber QP400-2-VIS-NIR (Ocean Insight), with core size of 400 µm, and detected with Ocean Insight USB4000-UV-VIS-ES. The gap between measurements was 1 second. A setup of lenses was used to enhance the signal (Scheme S1).

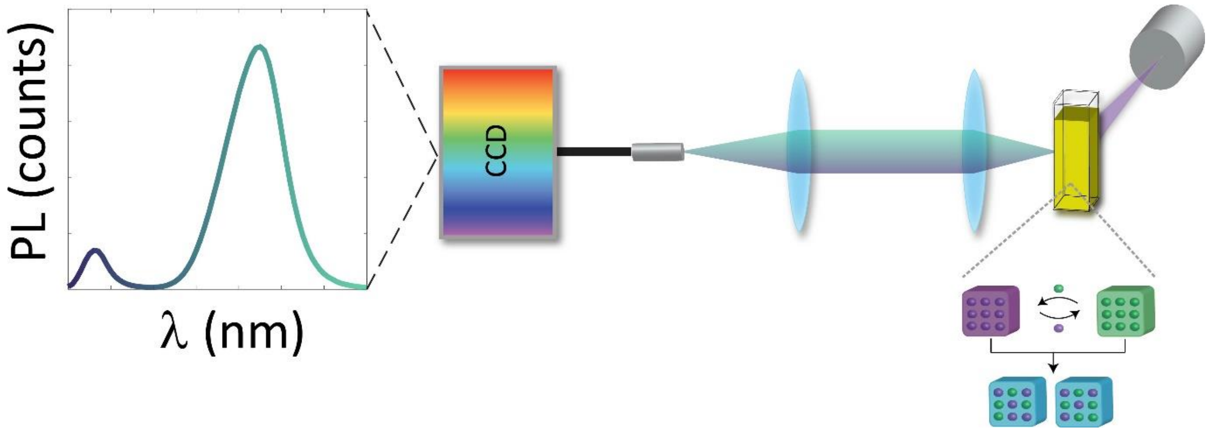

Scheme S1. Scheme of the setup used for light collection with an optical fiber and detection with a CCD detector. Focal length of lenses is 3 cm.

7. Anion exchange product

The final product of the cross-anion exchange reaction was further characterized by TEM and STEM. As presented in Figure S4, the NCs shape is preserved.

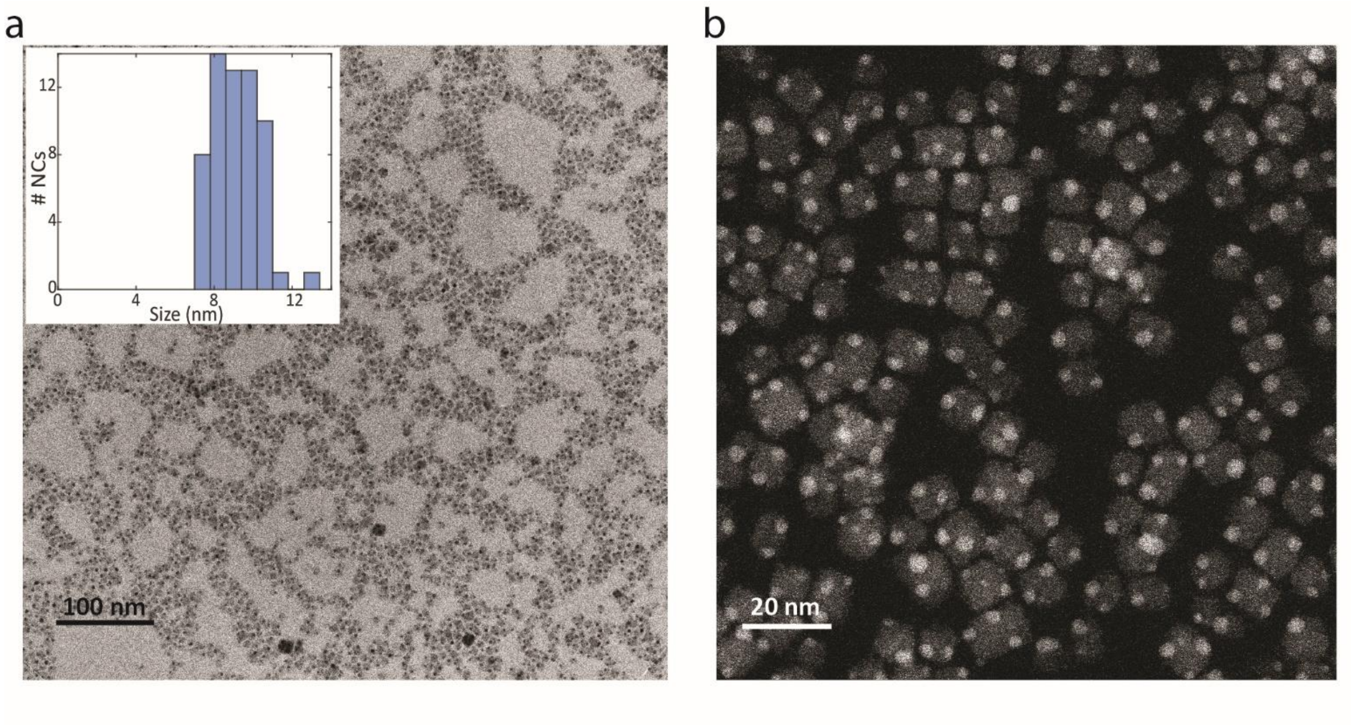

Figure S4. (a) TEM image of C3-ASC18 capped $CsPb(Cl_{0.9}Br_{0.1})_3$ after anion exchange. Inset: size distribution histogram of C3-ASC18 capped $CsPb(Cl_{0.9}Br_{0.1})_3$ after anion exchange with an average size of 9±1 nm. (b) Scanning transmission electron microscope (STEM) high-angle annular dark-field (HAADF) image of C3-ASC18 capped $CsPb(Cl_{0.9}Br_{0.1})_3$ after anion exchange.

8. Data analysis

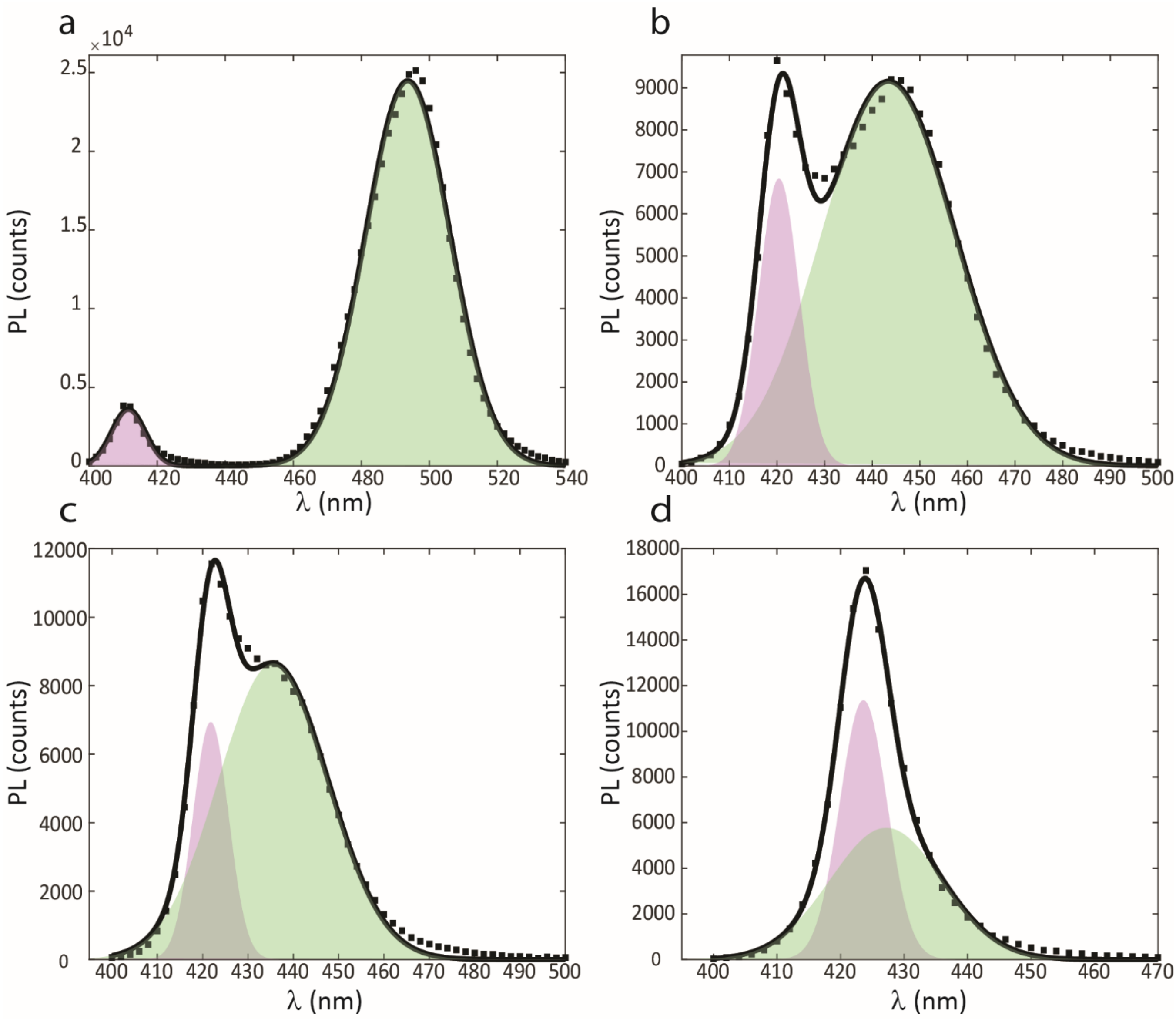

Figure S5. A sum of two Gaussians fit to the spectra during cross-anion exchange between C3-ASC18 capped $CsPbCl_3$ and C3-ASC18 capped $CsPbBr_3$ at 10 °C. (a) Spectra measured at t=30 sec (b) t=6 min, (c) t=8.3 min, (d) t=13.3 min.

Table S1. Rate constants of cross-anion exchange in the high energy peak and low energy peak of lecithin, 3-(N,N-dimethyl(octadecyl)ammonio)propane-1-sulfonate (C3-ASC18), and oleic acid/oleylammonium (OA/OLA) capped NCs at 25 °C.

High/low energy peak	Lecithin	C3-ASC18	OA/OLA
High energy peak	0.003±0.001 s^{-1}	0.016±0.001 s^{-1}	0.032±0.001 s^{-1}
Low energy peak	0.002±0.001 s^{-1}	0.011±0.001 s^{-1}	0.031±0.001 s^{-1}

9. Initial rates method

According to the initial rates method, the order of the reaction can be determined under the assumption that at the beginning of the reaction, the rate depends on the initial concentration of the reactants.[7] Therefore, the reaction order is the slope of the plot of the logarithm of the initial rate versus the logarithm of the initial concentration, according to equation:

Equation S3. $$\log(v_0) = \alpha \log[Cl]_0$$

Where α is the order, and in this case, $[Cl]_0$ is the initial concentration of chloride ions (which is equal to 8.5 times the initial concentration of bromide ions).

In order to extract the reaction order as described, several experiments were conducted at various initial halide concentrations, while keeping the total volume identical, and 8.5:1 ratio between chloride and bromide ions.

a. Concentration calculation:

The initial concentration of chloride ions was calculated from the absorbance of the solution, which is proportional to the NCs concentration, with the consideration of the number of halides per NC (details in section 4).

b. Initial rate determination:

The initial rate was determined using a linear fit on the initial portion of the data. The slope is the initial rate.

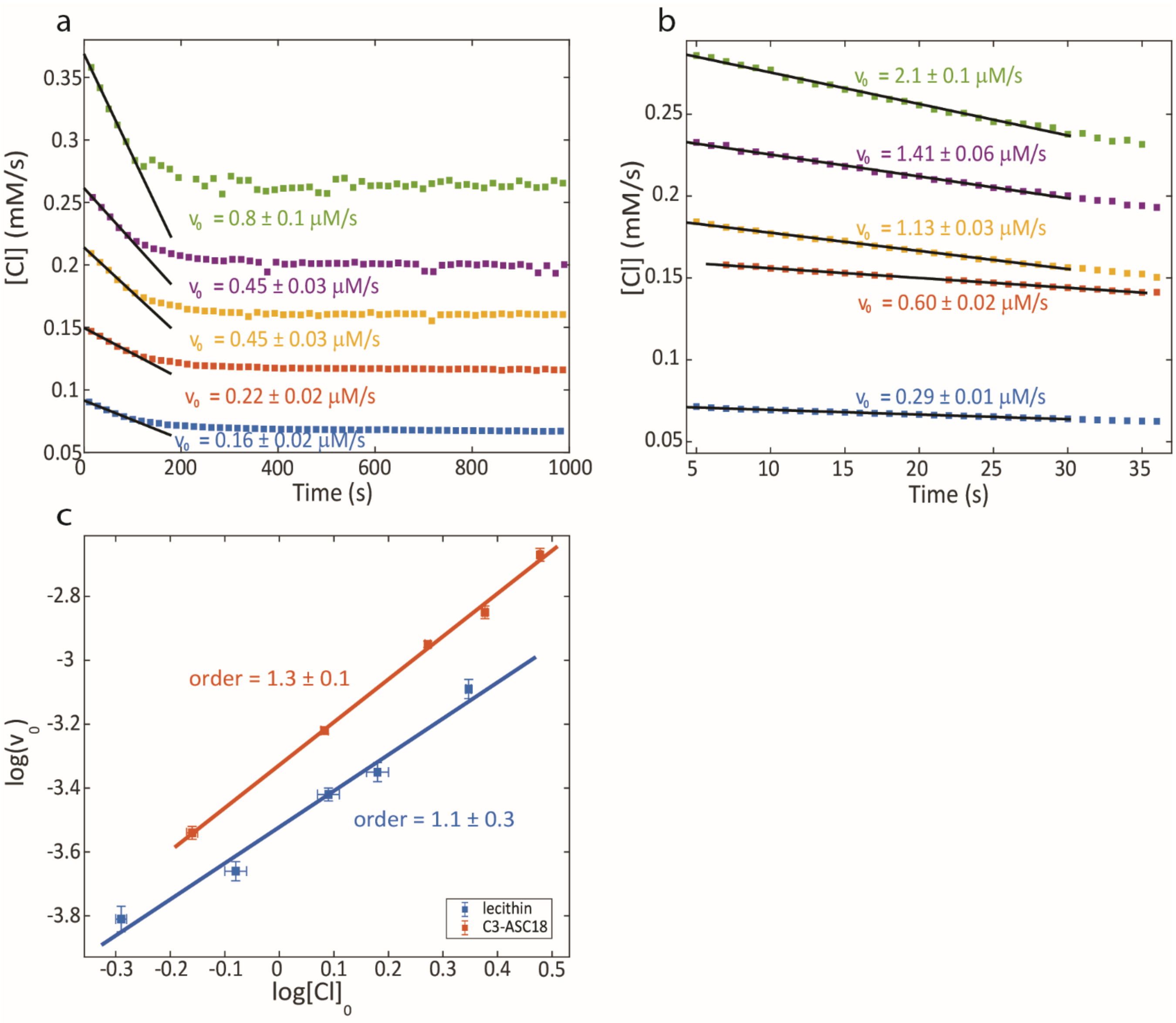

Figure S6. Linear fit at the beginning of the reaction, with a slope of the initial rate. (a) Chloride concentration versus time of lecithin capped NCs. (b) Zoom in to the linear portion of concentration versus time of C3-ASC18 capped NCs. (c) A plot of the logarithm of the initial rate versus the logarithm of the corresponding chloride ions concentration (8.5 times the bromide ions concentration), for determining the rates method.

10. Diffusion through a semipermeable membrane

The cross-anion exchange reaction was conducted in a setup comprising a semipermeable membrane allowing for the transfer of ligands but not of the NCs as demonstrated by a control of dialysis of NCs versus pure solvent (Figure S7). The PL was monitored at different times in the medium surrounding the dialysis bag, and at the end of the process, also for the medium inside it. The cross-anion exchange through the membrane was studied starting from pure solutions of C3-ASC18 capped $CsPbCl_3$ NCs within the dialysis bag, and of C3-ASC18 capped $CsPbBr_3$ NCs outside, with 8.5:1 halide ratio, respectively

The experiments were performed with C3-ASC18 capped $CsPbCl_3$ NCs solution inside a dialysis tube (Spectra/Por® dialysis membrane, Biotech CE tubing, MWCO: 1000 kD, width of 16 mm, and diameter of 10 mm), and $CsPbBr_3$ solution outside the tube.

a. Tube cleaning:

   As-purchased tubes are in a solution of 0.05% sodium azide in water. Since moisture is highly harmful to perovskite NCs, a cleaning procedure was necessary prior to the cross-anion exchange experiments.

   The tube was submerged in a solution of 15% ethanol in 1-propanol, and heated to 40 °C for 20 minutes. Then 5 ml of the solvent mixture was poured into the inner side of the tube. This step was repeated three times, and then three additional times with toluene at 60 °C.

b. Experimental setup:

   Control experiment: In order to confirm that the NCs cannot pass through the pores, we conducted a control experiment, in which a tube containing $CsPbBr_3$ NCs solution was immersed in pure toluene. After 3 hours the PL of the toluene

was measured and revealed no significant signal, which means that $CsPbBr_3$ NCs did not migrate through the membrane pores (Figure S7).

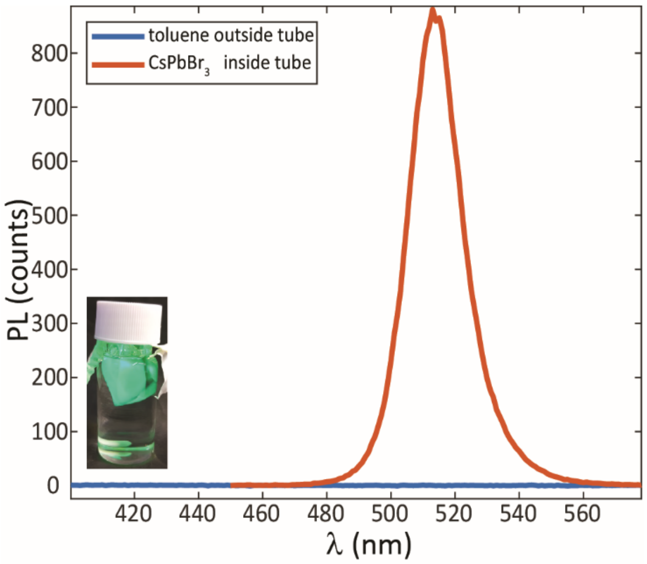

Figure S7. PL measurements at the end of the control experiment. Inset: the setup of anion exchange through a membrane.

The experiment was conducted in a vial with 19 ml of 0.05 μM $CsPbBr_3$ in toluene. Inside the tube was 2.6 ml of 0.3 μM $CsPbCl_3$ in toluene. Those quantities give an 8.5:1 ratio of chloride to bromide ions. The experiment was conducted for 7 hours. PL measurement of a $CsPbBr_3$ aliquot taken after 1.5 hours showed that the PL already started to blue shift. At this timescale the control experiment ruled out NCs crossing through the membrane (Figure S8).

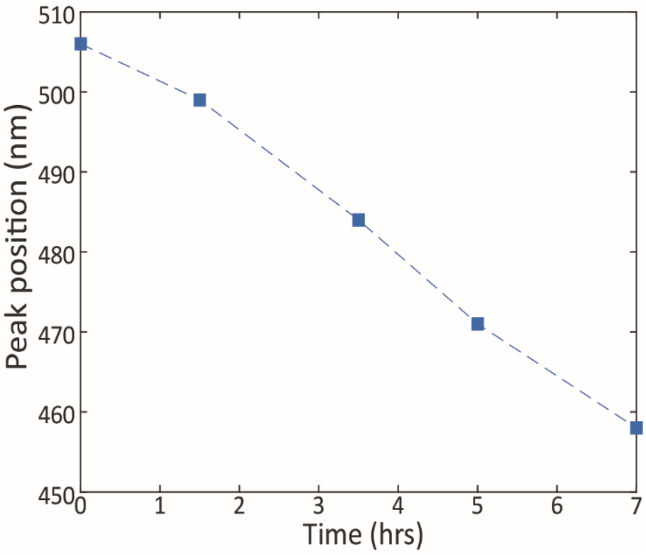

Figure S8. Peak position of $CsPbBr_3$ solution during cross-anion exchange through a semipermeable membrane.

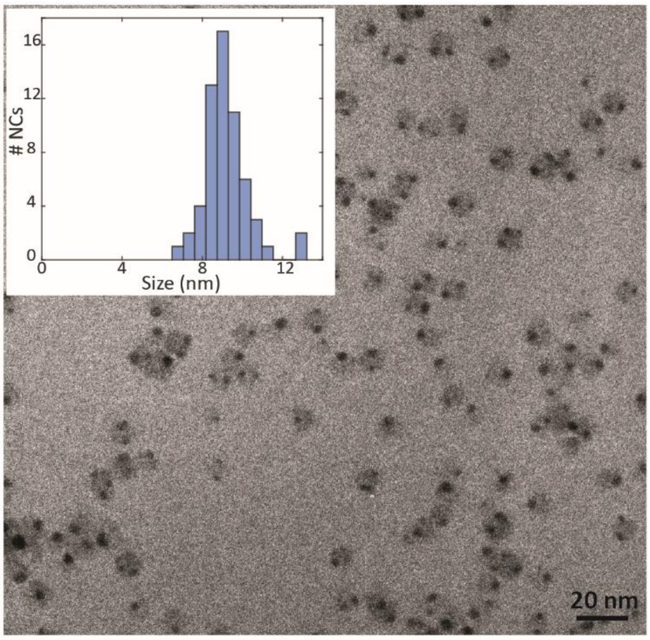

Figure S9. TEM image of the NCs after 7 hours of anion exchange through a semipermeable membrane. Inset: size distribution histogram of the product of anion exchange through the membrane with an average size of 9±1 nm.

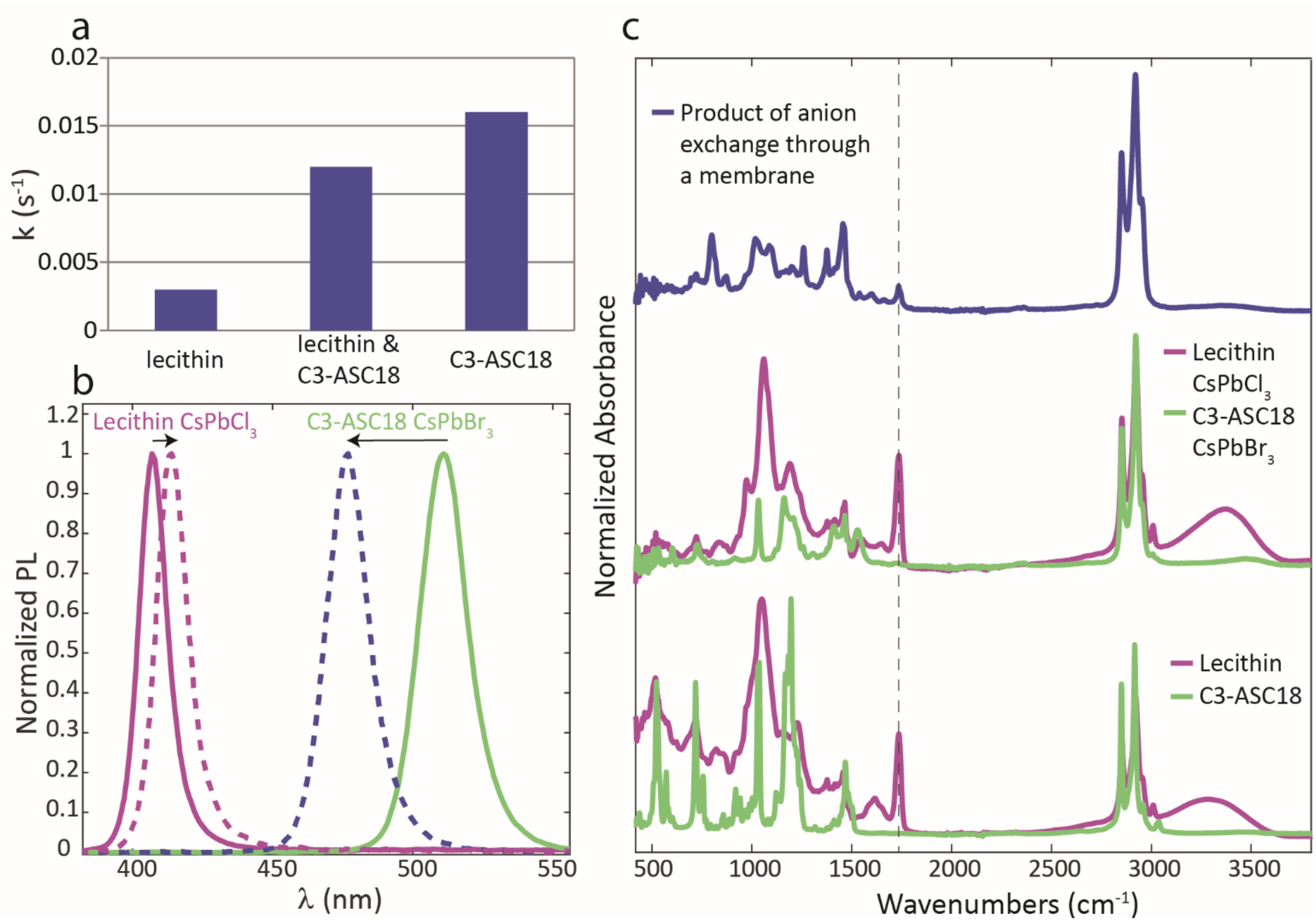

Figure S10. Results of cross-anion exchange between $CsPbCl_3$ capped with lecithin and $CsPbBr_3$ capped with C3-ASC18. (a) Rate constants achieved by kinetic experiments. (b) PL spectra of the $CsPbCl_3$ (purple) and $CsPbBr_3$ (green) NCs from inside/outside the dialysis bag at time t=0 (solid lines), and at time t=7 h (dashed lines) after the NCs were left to react through the membrane. (c) FTIR spectra of lecithin and C3-ASC18 (bottom panel), and of lecithin capped $CsPbCl_3$ and C3-ASC18 capped $CsPbBr_3$ (middle panel). The top panel is the FTIR spectrum of the solution in the side of the membrane that originally contained C3-ASC18 $CsPbBr_3$ (with the dashed dark purple spectrum in panel b). The C=O stretching peak at 1735 cm^{-1} showcases the diffusion of lecithin through the membrane.

11. Temperature effect

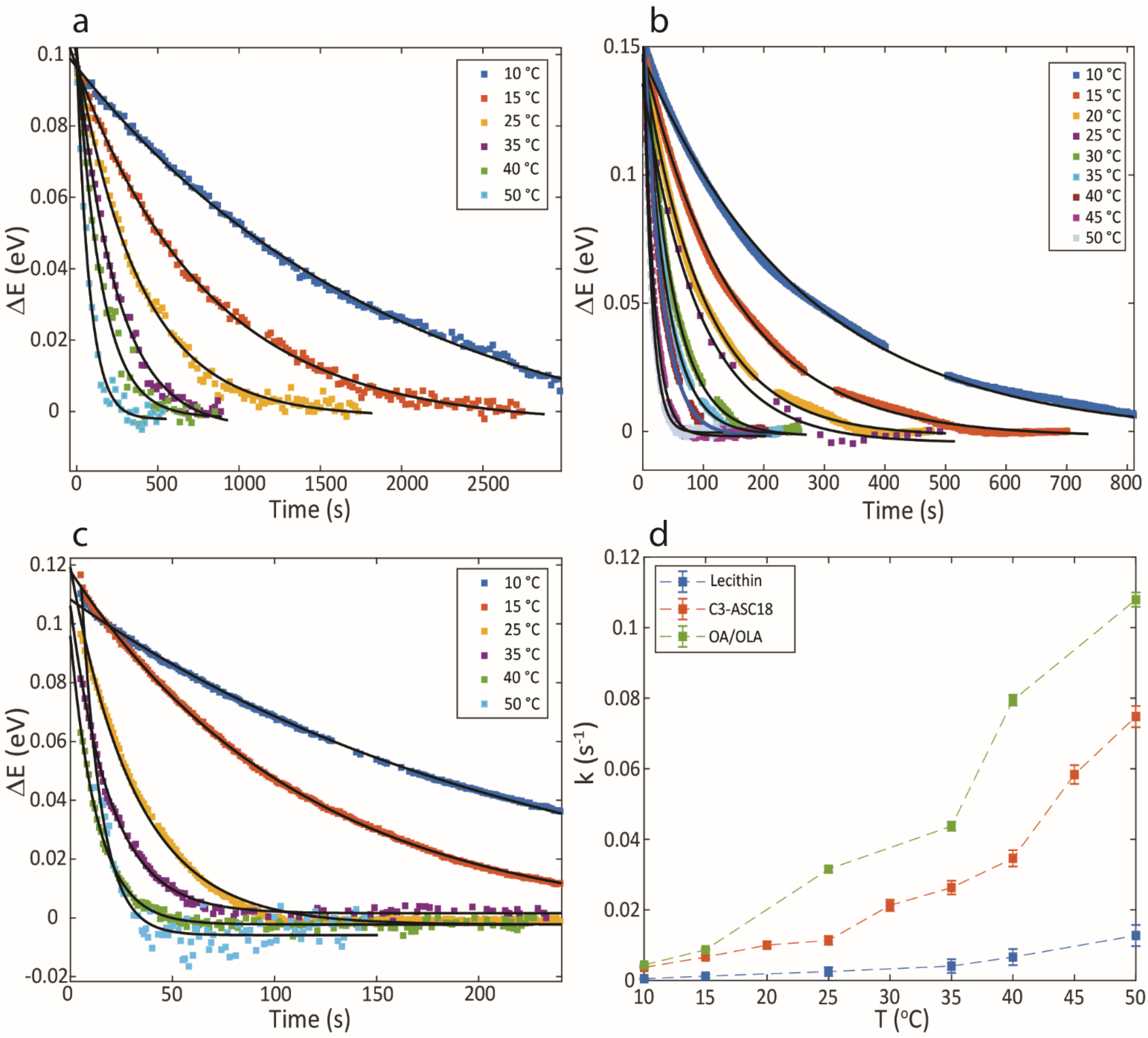

Figure S11. Exponential fitting to the difference between the energy at the emission peak and the energy at equilibrium versus time in different temperatures, of (a) lecithin capped NCs, (b) C3-ASC18 capped NCs, and (c) OA/OLA capped NCs. (d) The dependency of the rate constant on the temperature in lecithin, C3-ASC18, and OA/OLA capped NCs.

12. Size dependency

The kinetics of cross-anion exchange reaction depends on the size of the NC. However, the influence of the surface capping ligand is significantly stronger. Figure S12a presents the rate constants of the three systems, differing by the capping ligand, with similar sized NCs. According to the received trend, lecithin is the most stabilizing ligand and OA/OLA is the least stabilizing ligand over the studied size range. Figure S12b shows that the activation energy almost does not change with variation in the NC size, within the tested range.

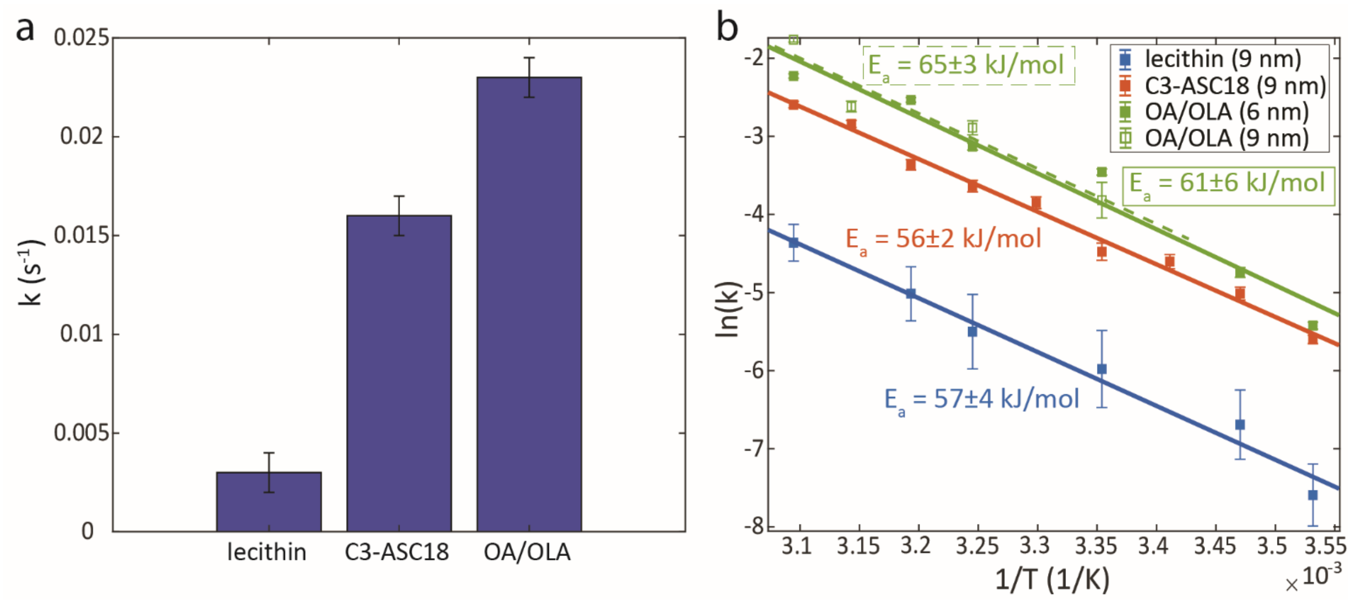

Figure S12. (a) Rate constants of cross-anion exchange in 9 nm NCs at 25 °C. (b) Arrhenius plot with a comparison between 6 nm OA/OLA capped NCs and 9 nm OA/OLA capped NCs. The green full squares and full line represent the 6 nm NCs, with an activation energy of 61±6 kJ/mol. The green empty squares and dashed line represent the 9 nm NCs, with an activation energy of 65±3 kJ/mol.

13. Mechanism

The investigated cross-anion exchange reaction can be written as:

Reaction S1. $$0.9CsPbCl_3 + 0.1CsPbBr_3 \rightarrow CsPb(Cl_{0.9}Br_{0.1})_3$$

Where a stoichiometric ratio of 8.5:1 chloride to bromide anions is considered. For simplicity, we refer to $CsPbCl_3$ and $CsPbBr_3$ NCs as $(NC)_{Cl}$ and $(NC)_{Br}$, respectively. For either pure NCs solution or during the cross-anion exchange, a ligand association and dissociation process is assumed to be in a pre-equilibrium condition:

Reaction S2.1 $$NC_{Cl} - L \rightleftarrows NC_{Cl} + L - Cl$$

Reaction S2.2 $$NC_{Br} - L \rightleftarrows NC_{Br} + L - Br$$

As the experiment cannot distinguish between sites that are bound to a ligand and sites that are not in the following reactions, NC-X describes a general surface site (X=Cl, Br).

The cage effect occurs between a surface site and a halide bound ligand

Reaction S3. $$NC - Cl + L - Br \underset{k_{-1}}{\overset{k_1}{\rightleftarrows}} NC - Cl \cdots Br - L \overset{k_2}{\rightarrow} NC - Br + L - Cl$$

The rate of the reaction can be expressed using the rate of product formation:

Equation S5. $$v = k_2[NC - Cl \cdots Br - L]$$

By using Steady state approximation on the concentration of the caged complex:

Equation S6. $$\frac{d[NC-Cl\cdots Br-L]}{dt}$$

$$= k_1[NC - Cl][L - Br] - k_{-1}[NC - Cl \cdots Br - L] - k_2[NC - Cl \cdots Br - L] = 0$$

the concentration of the caged complex is:

Equation S7. $$[NC - Cl \cdots Br - L] = \frac{k_1[NC-Cl][L-Br]}{k_{-1}+k_2}$$

The rate of the reaction, after plugging eq. (S10) into eq. (S8) is:

Equation S8. $$v = \frac{k_1 k_2[NC-Cl][L-Br]}{k_{-1}+k_2}$$

Considering the constant concentration of the free ligands, the reaction is a pseudo first order reaction with respect to the halide concentration.

Equation S9. $$v = k_{eff}[NC - Cl]$$

14. Viscosity measurements

Viscosities measurements were performed using Brookfield DV-II viscometer. (100 RPM).

Table S2. Viscosities of mixtures in different ratios of toluene and octadecene (ODE).

ODE fraction	Viscosity (cP)
0%	0.89±0.02
15%	1.07±0.02
35%	1.37±0.02
50%	1.59±0
60%	1.80±0.03
90%	3.23±0.03

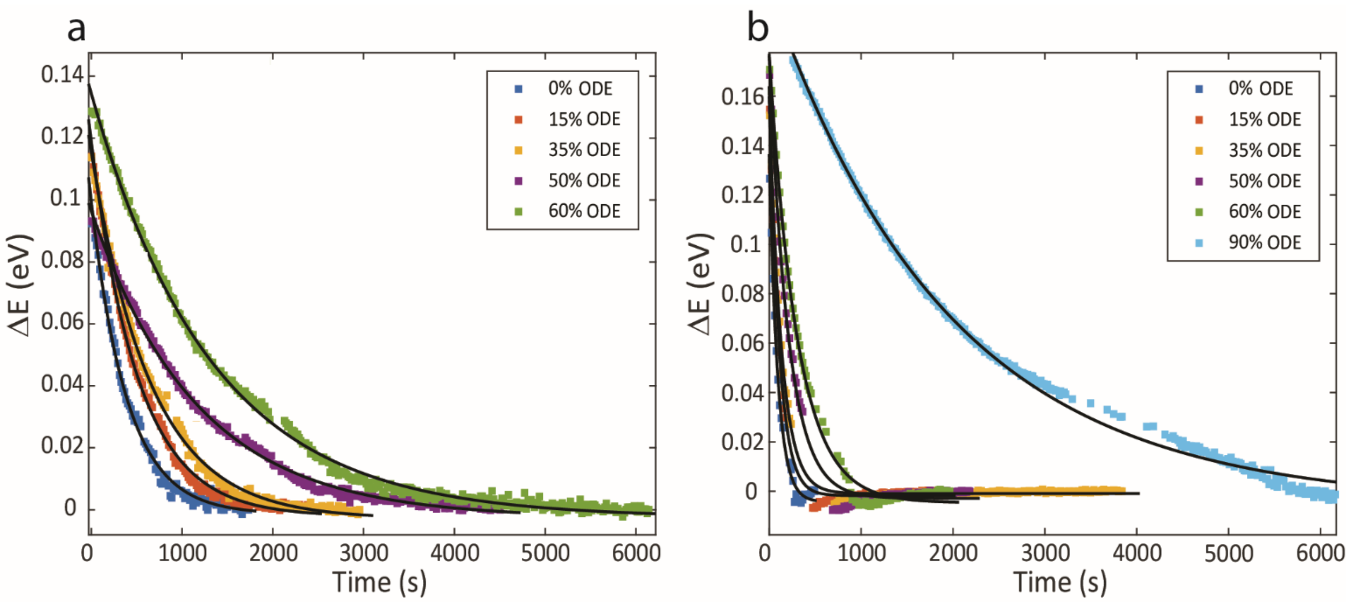

Figure S13. Exponential fitting to the difference between the energy at the emission peak and the energy at equilibrium versus time in various solution viscosities, of (a) lecithin capped NCs, and (b) C3-ASC18 capped NCs.

15. Surface coverage

a. Thermogravimetric analysis (TGA):

TGA enables to find the mass of the organic capping ligands and of the inorganic NCs, and consequently calculate the surface coverage percentage. Our calculation was with respect to Cs surface sites.

Equation S10.
$$surface\ coverage\ \% = \frac{n_{ligands}}{n_{surface\ sites}} \cdot 100\% = \frac{\frac{m_{ligands}}{Mw_{ligands}}}{\frac{m_{NCs}}{m_{NC} \cdot N_{Cs\ surface\ sites} \cdot N_{AV}}} \cdot 100\%$$

Where $m_{ligands}$ and m_{NCs} are extracted from the TGA thermogram, m_{NC} is calculated from the size and density (section 4a), and the calculation of $N_{Cs\ surface\ sites}$ is demonstrated in section 4b.

b. Excess lecithin:

A stock solution of lecithin in toluene was prepared. For each data point, with different ratio of lecithin per Cs surface sites, different volumes were added to the pure $CsPbCl_3$ NCs solution prior to the injection of the $CsPbBr_3$ NCs.

The ratio between lecithin and Cs surface sites:

Equation S11.
$$\frac{n_{ligand}}{n_{Cs\ surface\ sites}} = \frac{n_{capping\ ligands}+n_{added\ ligands}}{n_{Cs\ surface\ sites}} = \frac{\frac{c_{NCs}\cdot N_{Cs\ surface\ sites}\cdot(surface\ coverage)}{V_{NCs}}+c_{added\ ligand}\cdot V_{added\ ligand}}{N_{Cs\ surface\ sites}\cdot c_{NCs}\cdot V_{NCs}}$$

C_{NCs}, the concentration of the NCs, is calculated from the absorbance, according to Beer-Lambert law. The calculation of $N_{Cs\ surface\ sites}$ is demonstrated in section 4b. The surface coverage is a calculated from the TGA thermogram.

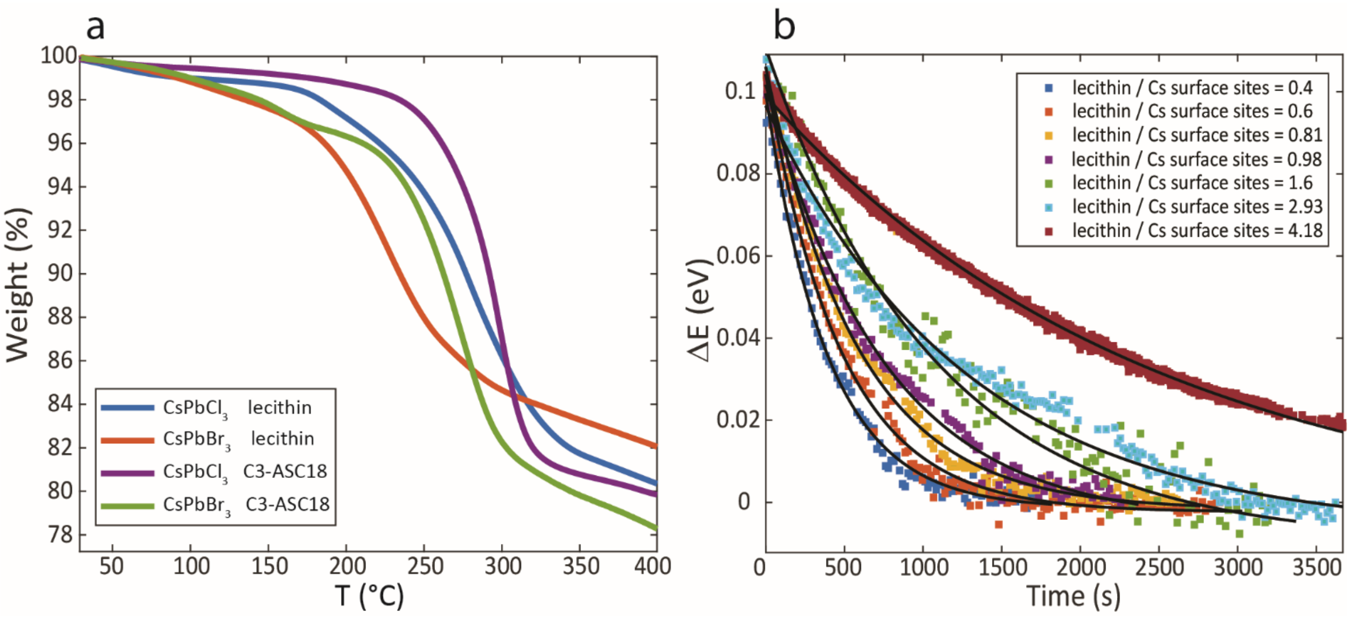

Figure S14. (a) TGA thermograms of NCs capped with lecithin and C3-ASC18. The calculation of the mass loss was in the range of 150-400 °C. The resulting surface coverage is: 40% for lecithin capped $CsPbCl_3$; 42% for lecithin capped $CsPbBr_3$; 63%

for C3-ASC18 capped $CsPbCl_3$; and 81% for C3-ASC18 capped $CsPbBr_3$. (b) Exponential fitting to the difference between the energy at the emission peak and the energy at equilibrium versus time, with varying ratios of lecithin per Cs surface sites.

References:

(1) Protesescu, L.; Yakunin, S.; Bodnarchuk, M. I.; Krieg, F.; Caputo, R.; Hendon, C. H.; Yang, R. X.; Walsh, A.; Kovalenko, M. V. Nanocrystals of Cesium Lead Halide Perovskites ($CsPbX_3$, X = Cl, Br, and I): Novel Optoelectronic Materials Showing Bright Emission with Wide Color Gamut. *Nano Lett.* **2015**, *15* (6), 3692–3696.

(2) Krieg, F.; Ochsenbein, S. T.; Yakunin, S.; Ten Brinck, S.; Aellen, P.; Süess, A.; Clerc, B.; Guggisberg, D.; Nazarenko, O.; Shynkarenko, Y.; Kumar, S.; Shih, C. J.; Infante, I.; Kovalenko, M. V. Colloidal $CsPbX_3$ (X = Cl, Br, I) Nanocrystals 2.0: Zwitterionic Capping Ligands for Improved Durability and Stability. *ACS Energy Lett.* **2018**, *3* (3), 641–646.

(3) Krieg, F.; Ong, Q. K.; Burian, M.; Rainò, G.; Naumenko, D.; Amenitsch, H.; Süess, A.; Grotevent, M. J.; Krumeich, F.; Bodnarchuk, M. I.; Shorubalko, I.; Stellacci, F.; Kovalenko, M. V. Stable Ultraconcentrated and Ultradilute Colloids of $CsPbX_3$ (X = Cl, Br) Nanocrystals Using Natural Lecithin as a Capping Ligand. *J. Am. Chem. Soc.* **2019**, *141* (50), 19839–19849.

(4) Nedelcu, G.; Protesescu, L.; Yakunin, S.; Bodnarchuk, M. I.; Grotevent, M. J.; Kovalenko, M. V. Fast Anion-Exchange in Highly Luminescent Nanocrystals of Cesium Lead Halide Perovskites ($CsPbX_3$, X = Cl, Br, I). *Nano Lett.* **2015**, *15* (8), 5635–5640.

(5) Maes, J.; Balcaen, L.; Drijvers, E.; Zhao, Q.; De Roo, J.; Vantomme, A.; Vanhaecke,

F.; Geiregat, P.; Hens, Z. Light Absorption Coefficient of $CsPbBr_3$ Perovskite Nanocrystals. *J. Phys. Chem. Lett.* **2018**, *9* (11), 3093–3097.

(6) Bertolotti, F.; Protesescu, L.; Kovalenko, M. V.; Yakunin, S.; Cervellino, A.; Billinge, S. J. L.; Terban, M. W.; Pedersen, J. S.; Masciocchi, N.; Guagliardi, A. Coherent Nanotwins and Dynamic Disorder in Cesium Lead Halide Perovskite Nanocrystals. *ACS Nano* **2017**, *11* (4), 3819–3831.

(7) Mortimer, M.; Taylor, P.; Smart, L. E.; Clark, G.; The Open University. *Chemical Kinetics and Mechanism*; Cambridge, UK: Royal Society of Chemistry, 2002, pp 60-62.
